# Thermo-Programmed Synthetic DNA-Based Receptors

**DOI:** 10.1021/acsnano.2c07039

**Published:** 2023-01-23

**Authors:** Davide Mariottini, Andrea Idili, Gianfranco Ercolani, Francesco Ricci

**Affiliations:** † Chemistry Department, University of Rome, Tor Vergata, Via della Ricerca Scientifica, 00133, Rome, Italy

**Keywords:** temperature-responsive nanocarriers, intrinsic disorder, entropy, molecular switches, DNA nanotechnology

## Abstract

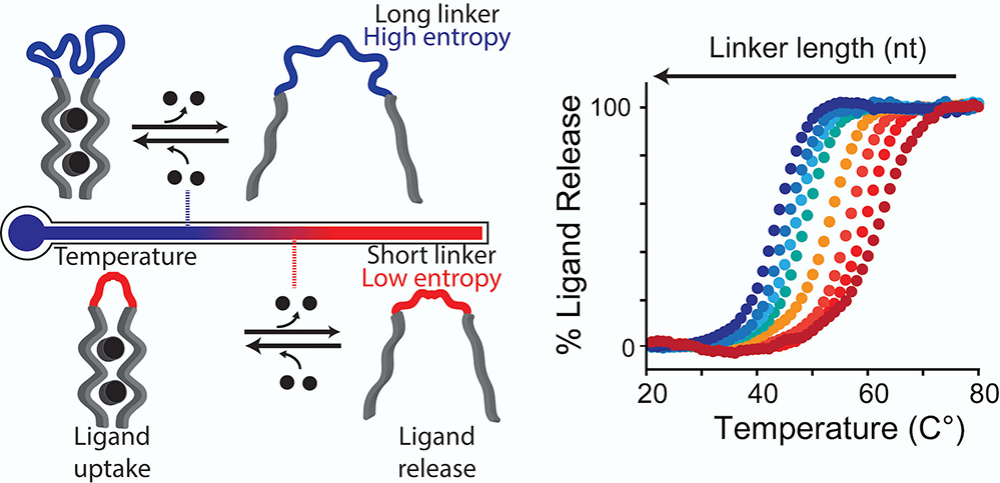

Herein, we present a generalizable and versatile strategy to engineer synthetic DNA ligand-binding devices that can be programmed to load and release a specific ligand at a defined temperature. We do so by re-engineering two model DNA-based receptors: a triplex-forming bivalent DNA-based receptor that recognizes a specific DNA sequence and an ATP-binding aptamer. The temperature at which these receptors load/release their ligands can be finely modulated by controlling the entropy associated with the linker connecting the two ligand-binding domains. The availability of a set of receptors with tunable and reversible temperature dependence allows achieving complex load/release behavior such as sustained ligand release over a wide temperature range. Similar programmable thermo-responsive synthetic ligand-binding devices can be of utility in applications such as drug delivery and production of smart materials.

## Introduction

Naturally occurring ligand-binding receptors are crucial to the functioning of life.^1−3^ Cell replication,^4,5^ sustainment, and reproduction^6^ mainly rely on receptors, such as enzymes and proteins, that recognize a specific ligand, load and release it to a target location, or use it to trigger a specific biological pathway and produce a certain metabolite. Nature has evolved an amazing variety of mechanisms with which the activity of such ligand-binding receptors can be finely modulated^7−9^ including the use of sophisticated allosteric mechanisms that allow controlling the load and release of a specific ligand in response to different molecular cues and environmental stimuli such as pH or temperature.^10,11^

Inspired by naturally occurring receptors, synthetic ligand-binding devices have emerged as versatile components of supramolecular tools that can find different applications including sensing and drug delivery.^12,13^ Among the different synthetic ligand-binding devices that have been developed so far, such as host–guest complexes,^14,15^ molecular imprinted polymers,^16,17^ and dendrimers,^18,19^ the use of synthetic oligonucleotides has emerged as particularly advantageous.^20−23^ Nucleic acid receptors can be in fact rationally designed to recognize and bind a specific complementary sequence through Watson–Crick–Franklin (W–C–F) interactions^24,25^ or, as in the case of aptamers, in vitro selected to recognize a small molecule or a protein.^26,27^ The programmability, predictability of interactions, and the chemical versatility of DNA allow finely modulating the binding affinity of these receptors in a highly controllable fashion using different stimuli with a precision similar to that of natural receptors. For example, inspired by allosterically regulated proteins, nucleic acid-based switches have been rationally designed to be controlled by different molecular activators or inhibitors.^28−33^ Similarly, taking advantage of the pH-dependence of Hoogsteen interactions,^34,35^ the affinity of DNA receptors has been also finely modulated using pH.^36−38^

Of note, the use of temperature as a way to dynamically control the functions (i.e., load or release of ligands) of DNA-based receptors has seen limited efforts. This appears surprising considering that the stability of W–C–F interactions is affected by temperature in a quite predictable fashion.^39^ This property has been successfully mainly exploited to engineer thermo-responsive DNA-based devices as programmable nanoscale thermometers.^40−44^ In other examples thermo-responsiveness of DNA–DNA interactions has been employed to control the assembly and functions of DNA-based nanostructures and receptors,^45−47^ to develop programmable DNA hydrogels,^48^ and to design DNA-based receptors operating out of equilibrium conditions.^49^ The availability of a rational approach to design programmable DNA-based receptors that can load and release a ligand at specific temperatures could thus extend the range of possible applications outside of thermal sensing.

**Figure 1:**
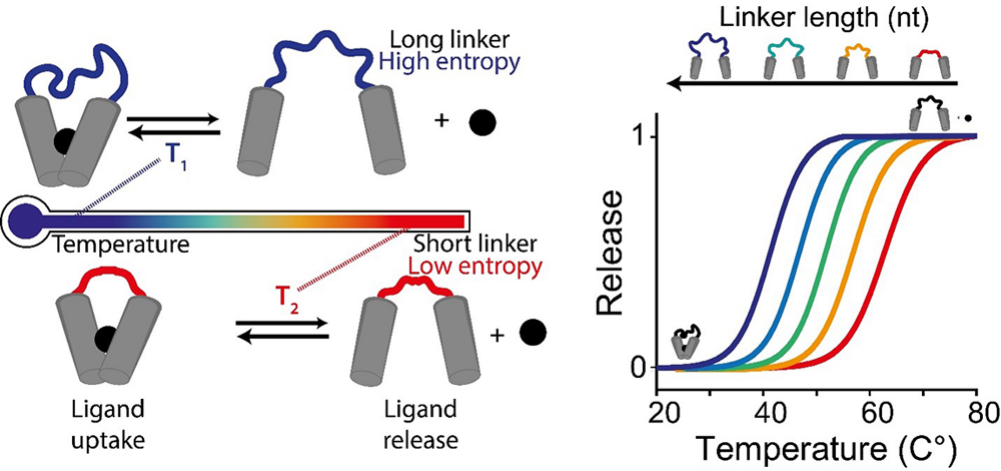
Thermo-regulated ligand-biding receptors. Thermo-responsiveness of a synthetic bivalent receptor can be finely modulated by controlling the length (and thus entropy) of the linker connecting the two binding domains. Receptors with longer linkers (associated with higher entropy) will release their ligand at a lower temperature compared to receptors displaying the same binding domains but with shorter linkers (associated with lower entropy). This provides a way to rationally program the temperature at which a synthetic receptor loads and releases its ligand.

Motivated by the above arguments, here we describe a strategy to design DNA-based thermo-regulated synthetic receptors. To do so, we took inspiration from naturally occurring bivalent receptors in which the ligand affinity can be controlled by intrinsically disordered domains (Figure 1).^50−52^ More specifically, we re-engineered bivalent ligand-binding DNA-based receptors in which the two binding domains are connected by an oligonucleotide linker. By controlling the length (and thus the entropy) of this linker we can finely modulate the temperature at which such synthetic ligand-binding receptors can load and release the ligand (Figure 1).

## Results and Discussion

**Figure 2:**
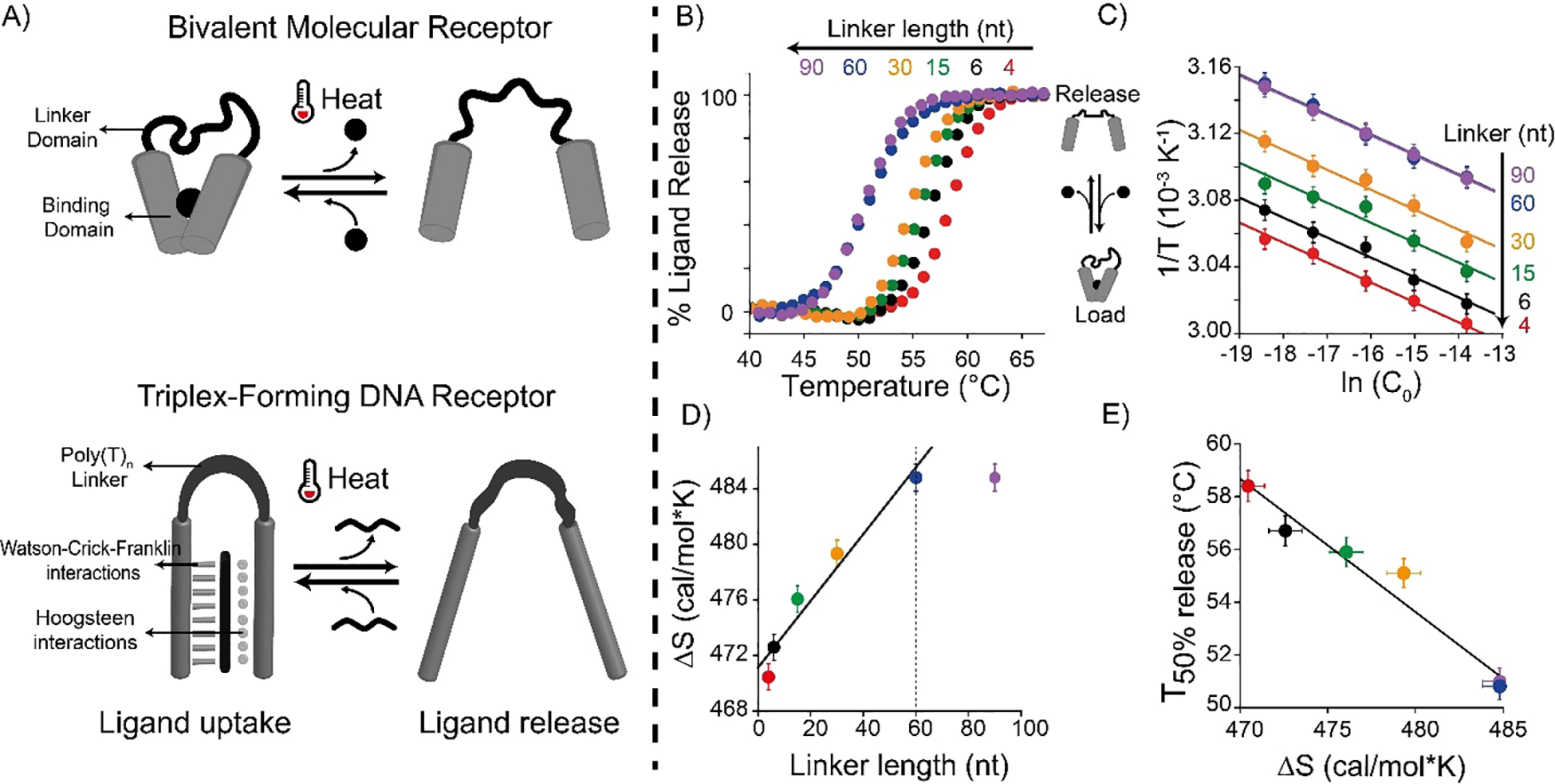
Rational design of a set of thermo-programmed triplex-forming DNA receptors. (A) (Top) Scheme of a classic bivalent molecular receptor in which two binding domains are connected by a linker domain. (Bottom) A synthetic DNA-based bivalent receptor in which two triplex-forming binding domains are connected by a poly-T linker domain. (B) Melting curves of DNA receptors sharing the same binding domains but with different lengths of the linker domain (indicated on the top of the panel). (C) 1/*T* vs ln(*C*_0_) plots obtained by thermal melting curves at different equimolar concentrations (*C*_0_) of DNA receptors and ligand. (D) Plot of Δ*S* vs linker length. (E) Plot of *T*_50%_ vs Δ*S* for the set of DNA receptors used. Melting curves experiments were performed in PBS buffer and 10 mM MgCl_2_ at pH 5.5 with a temperature ramp of 1 °C·min^–1^ and using a concentration of DNA receptor and ligand of 100 nM and 10 nM, respectively.

As our first test bed we have employed a synthetic DNA-based receptor directly inspired by naturally occurring bivalent receptors. Our synthetic DNA receptor contains two recognition domains (each of 13-nt, Figure 2A) joined by a polythymine (poly-T) oligonucleotide linker (gray, Figure 2A).^20,53,54^ The first domain binds a 11-nt DNA ligand through W–C–F interactions, forming a duplex, which then forms a DNA triplex structure with the second recognition domain through intramolecular Hoogsteen interactions. To control the thermo-responsiveness of this receptor, that is, the temperature at which the ligand is loaded and released, we engineered a set of bivalent receptors that share the same binding domains and differ in the length of the linker connecting them (i.e., from 4 to 90 thymines). Of note, we selected a poly-T linker due to its random coil behavior, which allows us to precisely introduce a purely entropic contribution.^55,56^ To characterize the loading/releasing process of the re-engineered bivalent receptors over a broad range of temperatures (Figure 2B), we labeled the ligand with a fluorophore/quencher pair: when the ligand is bound to the receptor, the fluorophore is forced away from the quencher and an increase in fluorescent signal is observed. Conversely, upon its release, the ligand folds into a random coil conformation bringing the fluorophore/quencher pair in closer proximity and leading to a decrease in fluorescent signal. Using melting experiments, we estimated for each receptor the *T*_50%_ value, i.e., the temperature at which the release of 50% of the ligand initially bound to the receptor is observed. Receptors with longer linkers progressively display lower *T*_50%_ values (Figure 2B and Table S1). For example, a receptor with a 90-nt linker displays a *T*_50%_ value of 51.0 ± 0.5 °C, which is 7.4 ± 1.0 °C lower than the *T*_50%_ given by the 4-nt linker receptor (see Table S1 and Figure S1). We observe a linear modulation of *T*_50%_ using linker lengths between 4-nt and 60-nt where, on average, each thymine added in the linker shifts the *T*_50%_ value of 0.12 ± 0.02 °C (see Figure S1). By further increasing the length of the linker (up to 90 nt) we do not observe further significant change in *T*_50%_ value, suggesting that a plateau in the modulation of thermo-responsive properties of the receptors has been reached (Figure S1). Control experiments using monovalent receptors (in which the triplex-forming portion is replaced with a random sequence unable to form the triplex structure) show, as expected, the same *T*_50%_ at different linker lengths (Figure S2 and Table S1), suggesting that the difference in *T*_50%_ values observed in the bivalent receptors is indeed due to the effect provided by the linker domain on the stability of the triplex structure.^53,54^

To better understand the role of the linker length on the modulation of the receptors’ thermo-responsiveness, we have experimentally determined the entropic contribution associated with each linker in the ligand-binding process. To do this, we performed additional melting curves at different concentrations (*C*_0_) of ligand and receptor (from 10 nM to 1 μM, in a 1:1 stoichiometric ratio) (see Figure S3 and Table S2). To estimate the enthalpic (Δ*H*) and entropic (Δ*S*) contributions associated with the load/release process, we plotted 1/*T*_50%_ values as a function of the natural logarithm of *C*_0_ (Figure 2C) (see SI).^57,58^ From the linear fits of these data we can extrapolate the enthalpic contribution associated with the formation of the triplex structure (slope) and its entropic content (intercepts).^57,58^ We found that all receptors display a similar enthalpic contribution (Δ*H* values within experimental error from each other; Figure S3 and Table S3). This is expected as the different receptors display the same recognition elements. The entropic values, conversely, increase linearly with the length of the poly-T linker (Figure 2D and Table S3). By plotting the entropic contribution for each receptor as a function of the poly-T linker length (Figure 2D), we estimated the entropic contribution provided by a single thymine introduced in the linker (i.e., 0.24 ± 0.03 cal·mol^–1^·K), a value that is in good agreement with previous studies using different DNA and RNA systems of comparable length.^54,59,60^ A linear relationship between the observed *T*_50%_ values and the calculated entropy values suggests that the observed modulation in thermo-responsiveness is driven by a purely entropic contribution associated with the linker (Figure 2E).

To verify how well the Δ*H* and Δ*S* values determined from the previous linear fits (Figure 2C and Table S3) reproduce the melting behavior of the receptors and the corresponding *T*_50%_ values, we obtained, by simple thermodynamic considerations, the theoretical expression for the fraction of saturated receptor as a function of the temperature (eq S9, see SI for further details). The accordance between the experimental melting curves for each receptor and those simulated by eq S9 is very gratifying (Figure S3), thus showing the consistence of the estimated enthalpic contribution and the entropy values associated with the different loops.

**Figure 3:**
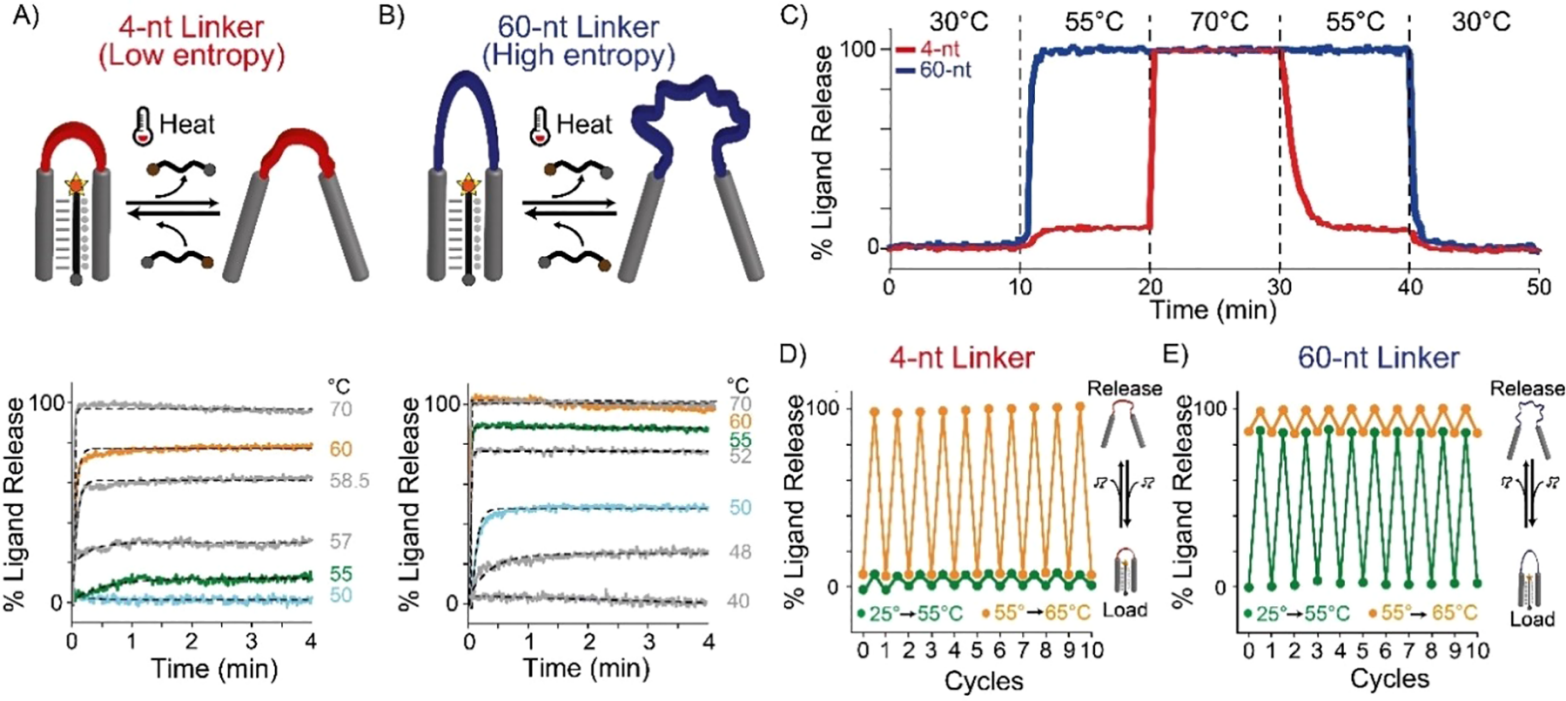
Thermo-programmed ligand’s load/release with triplex-forming DNA receptors. Release kinetics of triplex-forming DNA receptors with a 4-nt (A) and 60-nt (B) linker domain monitored through temperature jumps (from 25 °C to the indicated final temperatures). Three representative temperatures (50, 55, and 60 °C) were colored for better comparison. (C) Time-course experiments using two DNA receptors (4-nt in red and 60-nt in blue) in the same solution designed to bind two different 11-nt ligands (each labeled with a different fluorophore). (D, E) Load/release experiments at two temperature ranges (25 to 55 °C and 55 to 65 °C) for 4-nt (D) and 60-nt (E) triplex-forming DNA receptors. Time-course experiments (panels A and B) and load/release experiments (panels D and E) were performed in PBS buffer and 10 mM MgCl_2_ pH = 5.5 with a fixed concentration of DNA receptors (4-nt and 60-nt, 100 nM each) and 11-nt ligand (10 nM). For panel E two different DNA receptors were employed with two different 11-nt ligands.

Our approach allows achieving complex thermo-programmed load/release behavior by mixing DNA receptors with different linker lengths in the same solution. To demonstrate this, we employed two DNA receptors with short (4-nt) and long (60-nt) linkers each binding to a different ligand and labeled with a different fluorophore/quencher pair (A-488/BHQ1 and A-680/BHQ2), and we characterized their release kinetics at different temperatures (Figure 3A,B). Although each receptor releases its ligand over a different temperature range, both receptors show similar release kinetic constants (i.e., kt, min^–1^) at comparable % ligand release, suggesting that linker length does not affect the rate of the release process (Figure S4; see Table S4). We then performed in a solution containing both receptors a series of cyclic temperature jump experiments between three discrete temperature values: one at which no ligand release is observed for both receptors (30 °C), one where only one receptor (4-nt) releases its ligand (55 °C), and one at which both receptors release their ligands (70 °C) (Figure 3C). Doing this, we achieved differential release of the two ligands. For example, at 55 °C only the receptor with a 60-nt linker can fully release its ligand (blue line), while ∼90% of the second ligand remains bound to the other receptor (red line). By further increasing the temperature to 70 °C we can induce the complete release of the ligand from the second receptor. Because the loading/release process is reversible, by decreasing the temperature gradient we can achieve the gradual and differential reloading of the two ligands by the two receptors (Figure 3C).

The ligand-binding/release process is highly reversible, supporting multiple loading/release cycles. To demonstrate this, we performed load and release experiments using a real-time PCR thermocycler to achieve rapid temperature jumps from 25 to 55 °C (Figure 3D, green dots and lines) and from 55 to 65 °C (Figure 3E, orange dots and lines). The 4-nt and 60-nt receptors can release and load the ligand without losing their efficiency after 10 complete cycles.

We also demonstrate that the approach is versatile and can allow loading/releasing ligands of different lengths with the notable difference that this will occur over varying temperature ranges due to the different enthalpic contribution associated with the ligand/receptor binding (Figures S5 and S6).^53,61^ Similarly, as the triplex formation is strongly affected by pH and magnesium concentration,^53,62,63^ the temperature at which we can achieve control over the load/release process can also be modulated with these two experimental factors (Figure S7). Finally, the approach is also relevant for RNA sequences that can form triplex structures in a similar way to DNA sequences (Figure S8).^64−66^

**Figure 4:**
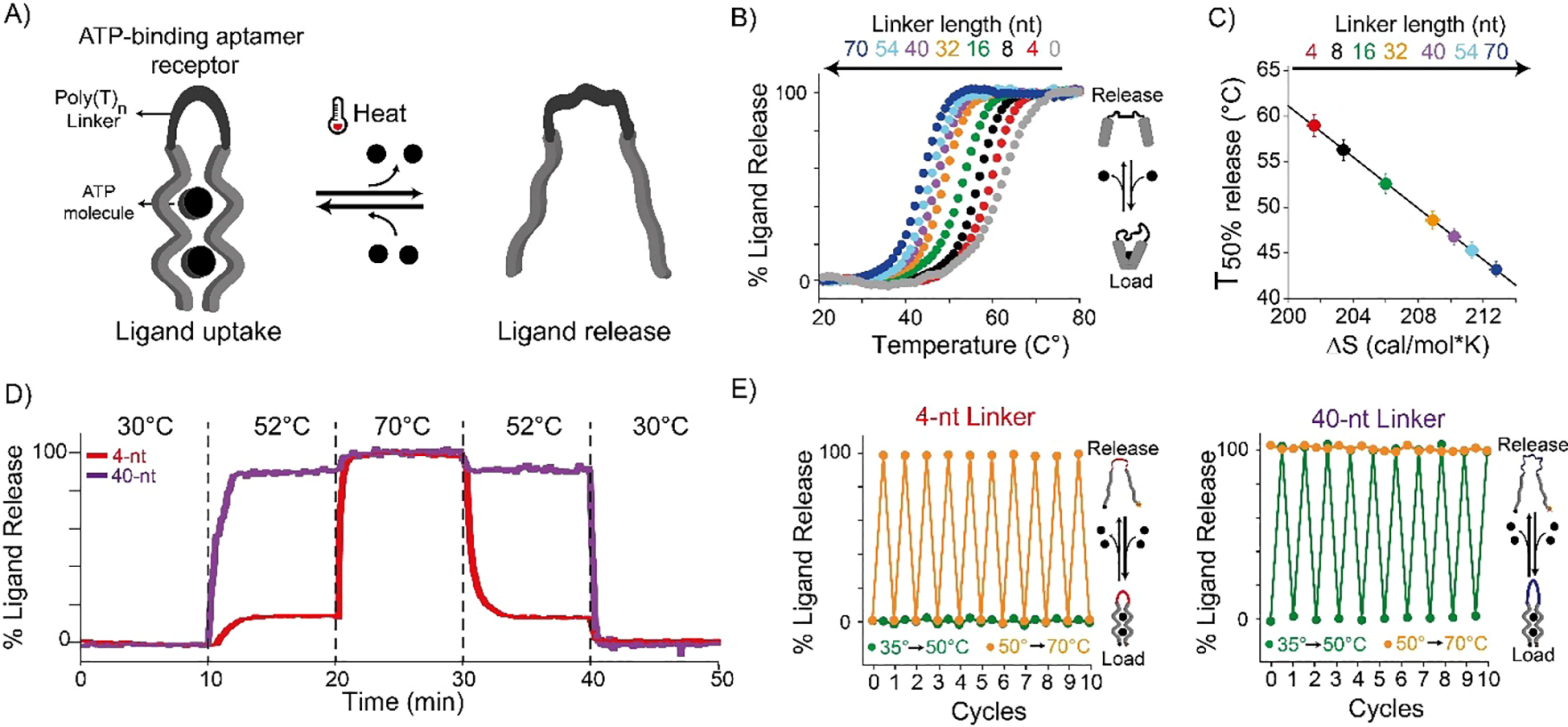
Thermo-programmed ATP-binding aptamer. (A) We re-engineered the ATP-binding aptamer to act as a bivalent-binding receptor by splitting its binding pocket and connecting them with a poly(T) linker domain. (B) Melting curves of ATP-binding receptors with different linker lengths. (C) Linear dependence between *T*_50%_ and Δ*S* values for the different receptors. (D) Time-course experiments obtained using two aptamer variants (4-nt in red and 40-nt in purple) in the same solution designed with the same binding domains for ATP but labeled with different fluorophores. (E) Load/release experiments at two temperature ranges (35 to 50 °C and 50 to 70 °C) for 4-nt and 40-nt ATP-binding aptamers. Melting curves experiments (B), time-course experiments (D), and load/release experiments (E) were performed in 100 mM Tris HCl and 10 mM MgCl_2_ at pH 6.5 with a ramp of 1 °C·min^–1^, with a fixed concentration of ATP-binding aptamer of 50 nM and 3 mM of ATP.

To demonstrate the versatility of our strategy and show that this can be used to thermally control other DNA-based receptors in a controllable way, we have re-engineered the ATP-binding aptamer, a well-characterized 27-nt DNA aptamer reported to bind two ATP molecules.^67−71^ We have split the native ATP-binding aptamer into two recognition domains that are connected by a poly-T domain of varying length (from 4 to 70 thymines) (Figure 4A). To monitor the loading/release of the ATP molecules, we labeled the aptamer sequence at the two ends with a fluorophore/quencher pair. Upon binding ATP, the aptamer folds into a closed-hairpin conformation,^68^ bringing the fluorophore and quencher in proximity and resulting in a suppression of the fluorescence signal. We show here that the length of the poly-T linker domain, and thus its associated intrinsic disorder, allows the fine control of the thermal responsiveness of ATP loading/release of the aptamer (Figure S9). To demonstrate this, we performed melting curves at a fixed concentration of ATP (3 mM) for all the ATP-binding split-aptamer variants (50 nM) (Figure 4B and Table S5). By varying the length of the linker domain from 4 to 70 nucleotides we modulate the *T*_50%_ values from 59.0 ± 0.5 to 43.2 ± 0.5 °C (see Table S5). In this case the addition of one thymine in the loop shifts the release of the ligand by an average of 0.23 ± 0.03 °C (Figure S9). This value is about 2-fold higher than that observed with the triplex-forming DNA receptor, a difference that might originate from the different binding mechanisms and structural features between the ATP-binding aptamer and the triplex-forming DNA receptor.

Also for the ATP-binding aptamer receptors we aimed at better understanding the role of the linker length by a more careful thermodynamic characterization of the binding event. In this case we could not follow the same approach employed with the triplex-forming receptor, as the ATP ligand needs to be added in large excess compared to the receptor. For this reason, we developed an ad-hoc model to determine the entropic contribution for the different variants, based on the following thermodynamic considerations. Let *y* be the fraction of free receptor, and *C*_L_ the concentration of excess ATP ligand; then, assuming an all-or-nothing cooperative binding of the ATP ligand, the dissociation constant of the ATP-saturated receptor is given by *K*_D_ = *yC*_L_^2^/(1 – *y*) (see SI for further details). Since from thermodynamics *K*_D_ = exp(−Δ*H*/*RT*)·exp(Δ*S*/*R*), equating the two expressions for *K*_D_ and solving for *y* yields the following equation relating the fraction of free receptor to temperature:
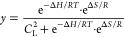
The experimental melting curves of ATP-binding receptors were fitted by nonlinear least-squares to the above equation, leaving Δ*H* and Δ*S* as adjustable parameters. Since, as expected, the obtained Δ*H* values were equal within the experimental errors (Table S6), to reduce the error of the estimated Δ*S* values, we fitted again the experimental melting curves to the above equation by fixing Δ*H* at its average value and leaving only Δ*S* as an adjustable parameter (Table S6). A very good fit of the melting curves (calculated using the average Δ*H* value and the Δ*S* values from Table S6) to the experimental points was obtained (Figure S10). By plotting the so-determined Δ*S* values as a function of the poly-T linker length (Figure S11), we estimated the entropic contribution provided by a single thymine in the linker as 0.16 ± 0.02 cal·mol^–1^·K, a value that is in substantial agreement with that of the triplex-forming DNA receptors (i.e., 0.24 ± 0.03 cal·mol^–1^·K, Figure 2D) and with previous studies using different DNA and RNA systems of comparable length.^54,59,60^ Also in this case, a very good linear correlation is observed between *T*_50%_ values and the obtained entropy values (Figure 4C) as expected for an entropy-driven thermo-responsiveness of the receptors.

Our approach allows achieving thermo-programmed aptamer receptors without affecting their load/release properties. To demonstrate this, we then selected two ATP-aptamer variants with low and high entropic contribution (4-nt and 70-nt, respectively) and characterized their release kinetics at different temperatures (Figure S12). Also in this case, the kinetic constants for the release process (i.e., kt, min^–1^) are not affected by the linker length at comparable ATP % release (see Figure S13, Table S7). We then performed a series of cyclic temperature jump experiments between three discrete temperature values (i.e., 30, 52, and 70 °C) in a solution containing the two ATP-binding aptamer variants labeled with different fluorophore/quencher pairs and demonstrated differential ATP release from the two receptors (Figure 4D). Cyclic load and release experiments performed using a real-time PCR thermocycler set over two different temperature jumps (from 25 to 55 °C and from 55 to 65 °C) show that the ATP thermal release by the two aptamer variants is fully reversible after 10 complete cycles (Figure 4E).

**Figure 5:**
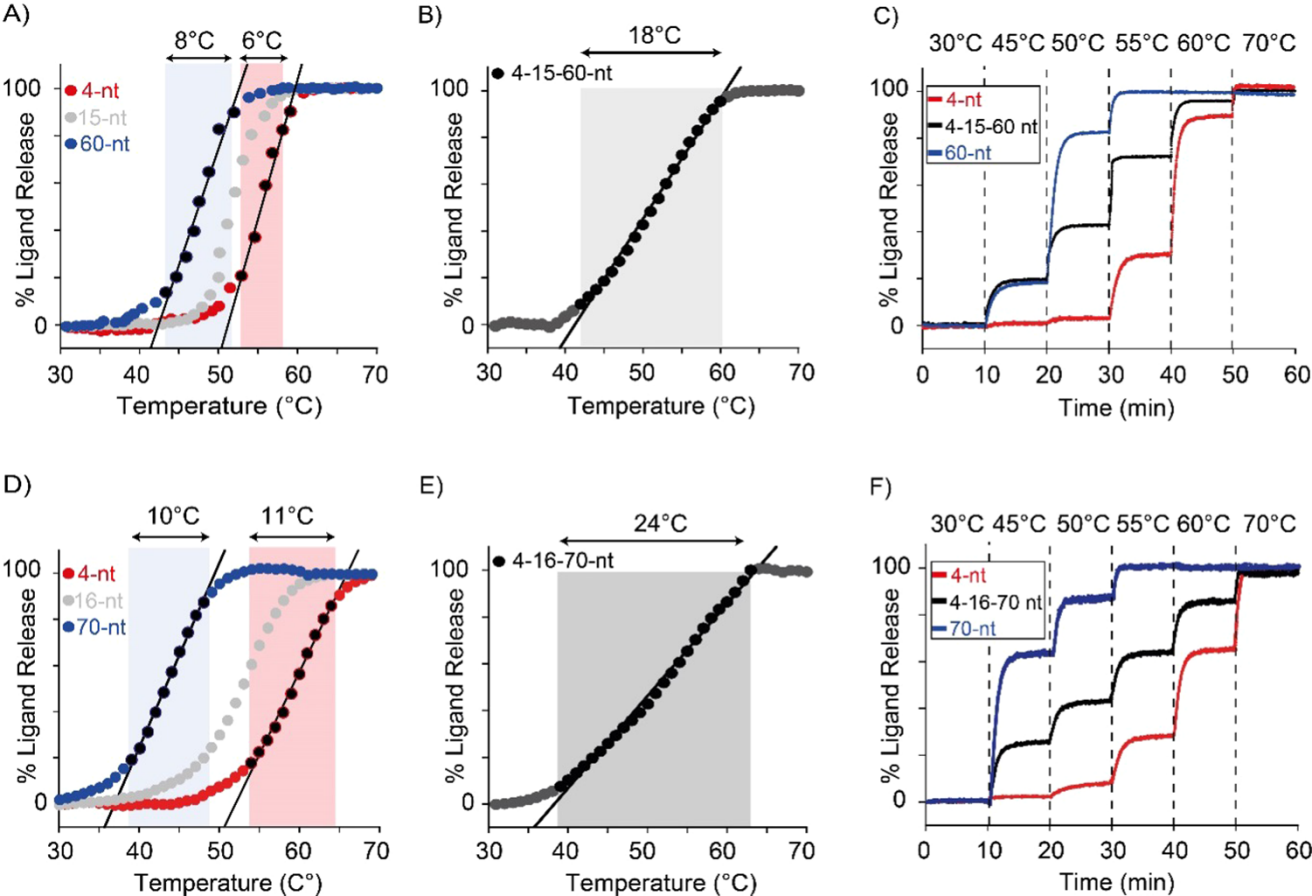
Extending the dynamic temperature range of ligand release. (A) Melting curves of triplex-forming DNA receptors (4-nt, 15-nt, and 60-nt linker) in the presence of ligand. (B) Extending the dynamic temperature range of ligand release by combining in the same solution a stoichiometric concentration of 4-, 15-, and 60-nt DNA receptors. (C) Time-course experiments at different temperature intervals for two receptors alone (4-nt, red and 60-nt, blue) and with three receptors (4-nt, 15-nt, and 60-nt) in the same solution (black). (D) Melting curves of ATP-binding aptamer variants (4-nt, 15-nt, and 60-nt linker) in the presence of ATP. (E) Extending the dynamic temperature range of ATP release by combining in the same solution a stoichiometric concentration of 4-, 16-, and 70-nt aptamer variants in the same solution. (F) Time-course experiments at different temperature intervals for two aptamer receptors alone (4-nt, red and 70-nt, blue) and with three variants (4-nt, 16-nt, and 70-nt) in the same solution (black). For triplex-forming DNA receptor melting curves (A), extended dynamic temperature range experiments (B), and time-course experiments (C) were performed in PBS buffer and 10 mM MgCl_2_, at pH 5.5 with a fixed concentration of DNA receptors (100 nM for a single receptor and 33.3 nM each for the mixture of three receptors) and 11-nt target (10 nM). For ATP-binding aptamer receptor melting curves (D), extended dynamic temperature range experiments (E) and time-course experiments (F) were performed in 100 mM Tris HCl and 10 mM MgCl_2_, at pH 6.5 with a fixed concentration of ATP-binding aptamers (50 nM for a single receptor and 16.6 nM each for the mixture of three receptors) and ATP (3 mM).

Programming the thermo-responsive properties of DNA synthetic devices by varying the length of the linker domain allows creating a set of receptors that bind to the same ligand but display different and controllable *T*_50%_ values. These receptors can be combined together to achieve a sustained ligand release over an extended temperature range. We first demonstrate this by using the triplex-forming DNA receptors. Each DNA receptor variant shows a temperature dynamic range (here defined as the range of temperature at which we observe a ligand release between 10% and 90%) that covers a fixed temperature interval of approximately 6–8 °C (Figure 5A). By mixing in the same solution three receptors with different linker lengths (4-, 15-, and 60-nt at equimolar concentration), we can extend such temperature dynamic range up to 18 °C (Figure 5B, black line). Time-course temperature jump experiments demonstrate how this approach can lead to a sustained release of the DNA ligand over a wider temperature interval compared to the single receptor (Figure 5C). This approach is generalizable, and we can apply it also to the ATP-binding aptamer variants described above. To do this we mixed three different ATP-binding aptamer variants (with linkers of 4-, 16-, and 70-nt; Figure 5E) and show that we can extend the fixed temperature dynamic range of the single aptamer from ∼10 °C to ∼24 °C (Figure 5D). Also in this case, time-course temperature jump experiments demonstrate how this approach can lead to a sustained release of ATP over a wide temperature range (Figure 5F).

## Conclusion

Here, we have proposed a generalizable and highly versatile strategy that allows programming with high precision the thermo-responsive properties of synthetic ligand-binding DNA-based receptors. To do this, we have rationally re-engineered a triplex-forming bivalent DNA-based receptor and an ATP-binding aptamer connecting their ligand-binding domains with a poly(T) linker. This domain is not involved in the ligand recognition event, and by controlling its length, and thus its entropy, we can finely control the temperature at which each receptor loads and releases its ligand. By doing so we demonstrate the possibility to create sets of DNA receptors with programmable temperature of ligand release that are reversible and rapid. The receptors can load or release ligands of different sizes, showing the possibility to shift the temperature range up to the physiological range. Additionally, these receptors can be combined together to achieve more complex thermo-responsive behavior and to provide sustained ligand release over a wide temperature range.

The proposed approach represents a highly programmable and generalizable strategy and could, in principle, be used to rationally tune the thermo-responsive properties of other synthetic nanodevices including those based on peptides and other polymers.^72−74^ We believe the proposed strategy could be used for a wide range of applications, including the design and assemblies of drug-delivery nanosystems with a finely tunable thermal dependence. A possible limitation in such applications could be represented by the presence of enzymes (e.g., helicase) that could interact with the receptors, affecting their load/release process. Compared to the current strategies exploited to modulate the delivery of a ligand in which the thermo-responsive properties are chemically controlled by changing the nature of the polymer or the component of the drug carrier,^72−74^ our approach appears advantageous because the entropy of the system can be gradually modulated through the length of the oligonucleotide linker. At the same time, this strategy could be employed to design and produce thermo-responsive materials, such as hydrogels^48,75^ or fibers,^76,77^ where the assembly of the material could be driven by supramolecular hierarchical mechanisms controlled by temperature.^72^

## Materials and Methods

### Chemicals

Unless otherwise stated we purchased all chemical reagents from Sigma-Aldrich (St. Louis, MO, USA), and we used them as received. These include phosphate-buffered saline (PBS tablets), magnesium chloride (MgCl_2_), hydrochloric acid (HCl), sodium hydroxide (NaOH), tris(hydroxymethyl)aminomethane hydrochloride salt (Tris·HCl), ethylenediaminetetraacetic acid (EDTA), and adenosine triphosphate (ATP).

### Oligonucleotides

HPLC-purified oligonucleotides were purchased from Metabion International AG, (Planegg/Steinkirchen, Germany) and Eurofins Genomics LLC (Louisville, KY, USA). All oligonucleotides were dissolved in TE buffer (10 mM Tris buffer, 1 mM EDTA, pH 7.8) at a concentration of 100 μM and frozen at −20 °C. RNA ligands were dissolved in DEPC water at a concentration of 100 μM and frozen at −20 °C. We estimated the concentration of the oligonucleotides measuring their relative absorbance at 260 nm using a Tecan Infinite M200pro (Männedorf, Switzerland) through a NanoQuant plate.

The sequences and the relative modifications of the receptors and ligands used are reported below.

Unlabeled synthetic DNA-based triplex-forming receptors:

Triplex-forming DNA receptor: 5′-TCCTTTCTCTCCT-(T)*_n_*-TCCTCTCTTTCCT-3′

Labeled synthetic DNA-based triplex-forming receptor:

Triplex-forming DNA receptor 4-nt loop: 5′-(ATTO495)-TCCTTTCTCTCCT-(T)_4_-TCCTCTCTTTCCT-(BHQ1)-3′

Here the underlined portion represents the triplex-forming domain and *n* represents the number of thymines of the linker domain.

Monovalent DNA-based triplex-forming receptors used for control experiments:

Triplex-forming DNA receptor 4-nt and 60-nt with random second recognition domain: 5′-CTTCGCTCTCATC-(T)_*n*_-TCCTCTCTTTCCT-3′

Labeled DNA ligands employed for the characterization of the triplex-forming receptors:

8-nt DNA ligand: 5′-(ATTO495)-T-AGGAAAGA-T-(BHQ-1)-3′

9-nt DNA ligand: 5′-(ATTO495)-T-AGGAAAGAG-T-(BHQ-1)-3′

10-nt DNA ligand: 5′-(AF488)-T-AGGAAAGAGA-T-(BHQ-1)-3′

11-nt DNA ligand: 5′-(AF488)-T-AGGAAAGAGAG-T-(BHQ-1)-3′

12-nt DNA ligand: 5′-(AF488)-T-AGGAAAGAGAGG-T-(BHQ-1)-3′

13-nt DNA ligand: 5′-(AF488)-T-AGGAAAGAGAGGA-T-(BHQ-1)-3′

Unlabeled RNA ligands employed for the characterization of the triplex-forming receptors:

10-nt RNA ligand: 5′-UAGGAAAGAGAU-3′

11-nt RNA ligand: 5′-UAGGAAAGAGAGU-3′

Ligand employed for the characterization of the triplex-forming receptors. Both ligands were used for time-course experiments using two DNA receptors in the same solution (Figure 3C).

11-nt DNA ligand #2: 5′-(ATTO680)-T-AAGAAGAAGGG-T-(BHQ-2)-3′

ATP-binding aptamer variants were modified with Alexa Fluor 488 (A-488) at the 5′ end and Black Hole Quencher 1 (BHQ-1) at 3′. ATP-binging aptamer 40-nt variant 2 used for time-course experiments using two aptamer receptors (Figure 4D) was modified with Cyanine 5.5 (Cy 5.5) at the 5′ end and Black Hole Quencher 2 (BHQ-2) at the 3′ end.

ATP-binding aptamer variants:

Native ATP-binding aptamer: 5′-(AF488) ACC TGG GGG AGT AT TGC GGA GGA AGG A(BHQ-1)-3′

Re-engineered ATP-binding aptamer: 5′-(AF488) ACC TGG GGG AGT AT-(T)n-TGC GGA GGA AGG A(BHQ-1)-3′

### Fluorescence Measurements

Melting curves and time-course experiments were obtained using a Cary Eclipse fluorimeter (Agilent Technologies) with an excitation wavelength at 490 (±5) nm and an acquisition wavelength at 517 (±5) nm for Alexa Fluor 488, an excitation wavelength at 520 (±5) nm and an acquisition wavelength at 525 (±5) nm for ATTO495, and an excitation wavelength at 680 (±5) nm and an acquisition wavelength at 702 (±5) nm for Alexa Fluor 680 (AF680). Finally, we used an excitation wavelength at 673 (±5) nm and an acquisition wavelength at 707 (±5) nm for 40-nt ATP binding aptamer variant #2 labeled with Cyanine 5.5 (Cy5.5). More details on the experimental procedures employed for temperature cycles and time-course experiments can be found in the Supplementary Methods.

### Melting Curve Experiments

Thermal melting curves were carried out using a fixed concentration of triplex-forming DNA receptor (100 nM) and 11-nt DNA ligand (10 nM) in PBS buffer (10 mM phosphate buffer solution, 137 mM NaCl, 2.7 mM KCl) with 10 mM MgCl_2_, at pH 5.5.

Thermal melting curves used for the characterization of the thermo-responsive properties of ATP-binding aptamer variants (Figure 4B) were carried out using a fixed concentration of ATP-binding aptamer (50 nM) and ATP (3 mM) in 100 mM Tris·HCl and 10 mM MgCl_2_, at pH 6.5.

Melting curves were performed by heating the solution containing the ligand and the receptor from 15 or 20 °C to 90 °C at a rate of 1 °C·min^–1^ using a total reaction volume of 1 mL in a quartz cuvette. To limit the evaporation of the sample during the experiment, a thin layer of mineral oil was added to the top of the solution.^57,58^ All the reported melting curves have been normalized using the interpolation model^57,58^ that allows estimating the temperature at which 50% of the ligand initially bound to the receptor is released (i.e., *T*_50%_ value). Specifically, the fluorescence intensity of the ligand/receptor complex in its loaded (*B*_L_, before melting transition) and released state (*B*_R_, after melting transition) has been chosen and fitted using a classic linear equation to obtain two baselines. By averaging the estimated baselines (upper and lower) over the temperature window tested, it is possible to calculate a median straight line that will be drawn within the two baselines, and it will cross the experimental curve in the middle of the melting transition region. This intersection point will correspond to the *T*_50%_ value, and its uncertainty is estimated at ±0.5 °C.^57,58^ Then, we converted the raw fluorescence signal collected at different temperatures (*F*_T_) to % ligand release using the following equation:
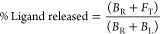
1where *B*_L_ and *B*_R_ correspond to the baseline values of the loaded and released state of the ligand/receptor complex, respectively, at different temperatures.

Further experimental details, thermodynamic analysis, ligand release kinetics, cyclic temperature jump, and load/release time-course experiments are given in the Supporting Information.

## Supporting Information Available

Thermodynamic analysis and theoretical models for the triplex-forming DNA receptors and the ATP-binding aptamers, ligand-release kinetics of DNA-based receptors, cyclic temperature jump experiments, load/release cycle, and supporting figures and tables (PDF)

## Notes

The preprint version of this paper is available on ChemRxiv: Mariottini, D.; Idili, A.; Ricci, F. Thermo-programmed synthetic DNA-based receptors. 2022, ChemRxiv. https://chemrxiv.org/engage/chemrxiv/article-details/62cfebbe4e76bfd7af8d7a36 (accessed January 18, 2023).
