# Supplementary material for: Thermo-Programmed Synthetic DNA-Based Receptors
Source: ACS Nano. 2023 Jan 23;17(3):1998–2006. doi: 10.1021/acsnano.2c07039 (PMC9933611; doi:10.1021/acsnano.2c07039)
Supplement: Supplementary file 1 — nn2c07039_si_001.pdf [file nn2c07039_si_001.pdf]

SUPPLEMENTARY INFORMATION

Thermo-programmed synthetic DNA-based receptors

*Davide Mariottini,[1, ‡] Andrea Idili,[1, ‡, *] Gianfranco Ercolani,[1] and Francesco Ricci[1, *]*

[1]Chemistry Department, University of Rome, Tor Vergata, Via della Ricerca Scientifica, 00133,

Rome, Italy.

I – EXPERIMENTAL SECTION

1.1 Thermodynamic analysis for the triplex-forming DNA receptors

For the thermodynamic analysis of the triplex-forming DNA receptors, we used a previously reported approach valid to characterize oligonucleotide-based bimolecular reactions.[1,2] Specifically, we have performed melting curves using 5 different concentrations of triplex-forming DNA receptor and 11-nt DNA ligand 1 in 1:1 stoichiometric ratio (10 nM, 30, nM, 100 nM, 300 nM and 1 μM) to cover a concentration range of about 100-fold [Puglisi and Tinoco, 1989; Mergny and Lacroix, 2003].[1,2] Considering C_0 value as the final concentration of the oligonucleotides involved in the experiments, which is given by the sum of the concentrations of the receptor and the ligand, we obtained a final C_0 of 10 nM, 30 nM, 100 nM, 300 nM, and 1 μM (see Figure S3 and Table S2). Plotting $1/T_{50\%}$ values as a function of the natural logarithm of the estimated C_0 values, we obtained a linear trend for each receptor that can be fitted by the following equation:[1,2]

$$1/T_m = \frac{\Delta S°}{\Delta H°} + \frac{\left(R ln\left(\frac{C_0}{2}\right)\right)}{\Delta H°} \qquad \text{(Eq. 1)}$$

This linear fit allows to estimate the enthalpy (ΔH) and entropy (ΔS) values associated to the loading and releasing process of the 11-nt DNA ligand 1 for each triplex-forming receptor, where the slope of the line corresponds to R/ΔH (R = 1,9872 $cal \cdot K^{-1} \cdot mol^{-1}$) while the intercept describes the ΔS. As expected, the estimated enthalpy contribution is almost the same for all triplex-forming receptors (i.e., they are within experimental error from each other) (see Table S3). To improve the precision of the estimation of ΔS values, we averaged the estimated enthalpic values obtaining a fixed ΔH value (165.9 ± 8.3 $kcal \cdot mol^{-1}$, Table S3) that can be used in Eq. 1 to calculate the

entropic contribution (ΔS) for each variant (see Table SI3). Moreover, to precisely estimate the entropic contribution associated to the different poly(T) linkers, we have used the triplex-forming receptor with the shortest linker (i.e., four nucleotides) as our reference, and we have subtracted its entropy from the entropic values estimated for the other receptors.

1.2 Theoretical model for the triplex-forming DNA receptors

In this work we developed a theoretical model to predict the thermo-responsive properties of the triplex-forming DNA receptors (Figure S3) using the enthalpic and entropic values (Table S3) estimated with the experimental procedure described in the previous section (Figure 2C and Table S3). Below we give a brief description of the rationale behind this model.

The melting transition of the receptor-ligand complex can be described as:

$$\mathrm{R{\bullet}L} \overset{K_d}{\rightleftharpoons} \mathrm{R} + \mathrm{L}$$

Where R is the receptor, L is the ligand, and K_d is the dissociation constant. This equilibrium gradually shifts towards the released (unfolded) state when increasing the temperature. Exploiting the relationship between the equilibrium constant and the free energy (ΔG) we can describe the K_d using the following equation:

$$K_d = e^{-\Delta H/RT} e^{\Delta S/R} \qquad \text{(Eq. 2)}$$

where ΔH is the enthalpy, ΔS is the entropy, T is the temperature, and R is the molar gas constant ($R = 1{,}9872\ \mathrm{cal{\cdot}K^{-1}{\cdot}mol^{-1}}$). Thus, exploiting ΔH and ΔS values experimentally obtained, the dissociation constant K_d can be estimated as a function of the temperature. To do this, we consider the concentrations of the receptors and the ligand used in the melting experiments (Figure S3) which are equimolar, leading to the following condition:

$$C_R = C_L = C \qquad \text{(Eq. 3)}$$

Where C_R is the concentration of the receptor, and C_L is the ligand concentration. Exploiting this condition, we can describe the molar concentrations in function of the variable x defined as the fraction of the reacted ligand and receptor and the constant C:

$$[R \cdot L] = C\,x \qquad \text{(Eq. 4)}$$

$$[L] = [R] = C\,(1 - x) \qquad \text{(Eq. 5)}$$

Using these conditions, we can express the K_d as follow:

$$K_d = \frac{[R][L]}{[R \cdot L]} = \frac{C(1-x)^2}{x} \qquad \text{(Eq. 6)}$$

Which can be converted to a second-degree equation and can be solved as follow:

$$Cx^2 - (K_d + 2C)x + C = 0 \qquad \text{(Eq. 7)}$$

$$x = \frac{(K_d + 2C) - \sqrt{(K_d + 2C)^2 - 4C^2}}{2C} \qquad \text{(Eq. 8)}$$

Then, substituting Eq. 2 into Eq. 8, we can describe the fraction of the reacted ligand and receptor in function of the thermodynamic values:

$$x = \frac{\left(e^{-\Delta H/RT} e^{\Delta S/R} + 2C\right) - \sqrt{\left(e^{-\Delta H/RT} e^{\Delta S/R} + 2C\right)^2 - 4C^2}}{2C} \qquad \text{(Eq. 9)}$$

Introducing in Eq. 9 the enthalpic and entropic values calculated through the melting experiments (Figure 2C and Table S3) and the relative concentration (equimolar) of the receptor and the ligand,

we estimated the value of the fraction x for each temperature which allow to simulate the melting curve for each receptor (Figure S3)

1.3 Theoretical model and thermodynamic analysis for the ATP-binding aptamer

For the characterization of the ATP-binding aptamer variants, we have developed an ad-hoc theoretical model which is used to: 1) analyze the melting curves of the ATP aptamer receptors and precisely estimate the ΔH and ΔS values (Table S6); 2) predict the thermo-responsive properties of the aptamer receptors (Figure S10). Below we give a brief description of the rationale behind this model.

The melting transition of the receptor-ligand complex can be described as:

$$R\bullet L_2 \underset{}{\overset{K_d}{\rightleftharpoons}} R + 2\,L$$

Where R is the aptamer receptor, L is the ATP ligand, and K_d is the dissociation constant. This equilibrium gradually shifts towards the released (unfolded) state when increasing the temperature. The K_d can be described as function of the free energy (ΔG) as reported n Eq. 2 and can be estimated as a function of the temperature. To do this, we need to describe the molar concentrations as function of the variable y defined as the fraction of free receptor and the constants C_R and C_L, leading to the following conditions:

$$[R] = C_R\, y \qquad \text{(Eq. 10)}$$

$$[L] = C_L - 2\, C_R\,(1 - y) \approx C_L \qquad \text{Since } C_L >> C_R \qquad [L] \approx C_L \qquad \text{(Eq. 11)}$$

$$[R{\cdot}L_2] = C_R\,(1 - y) \qquad \text{(Eq. 12)}$$

Where C_R is the concentration of the receptor, and C_L is the ATP ligand concentration. Because

the concentration of the ATP ligand is in large excess respect to the receptor, we can assume the molar concentration of the ligand as a constant. Using Eq. 11-13, we can describe the K_d as function of the fraction of the free receptor as follow:

$$K_d = \frac{[R][L]^2}{[R \cdot L_2]} = \frac{yC_L^2}{1-y} \qquad \text{(Eq. 13)}$$

This last equation can be related to the thermodynamics through Eq. 2, and we can describe the fraction of the free receptor in function of the thermodynamic values and temperature, as follow:

$$y = \frac{e^{-\Delta H/RT} e^{\Delta S/R}}{C_L^2 + e^{-\Delta H/RT} e^{\Delta S/R}} \qquad \text{(Eq. 14)}$$

The melting curves of ATP-binding receptors in the presence of excess ATP ligand (Figure 4B) have been fitted to Eq. 14 by nonlinear least-squares fit method. This fitting procedure allows to estimate the enthalpy (ΔH) and entropy (ΔS) values associated to the loading and releasing process of ATP ligand for each aptamer receptor (Table S6). Since, as expected, the obtained ΔH values were equal within the experimental errors (Table S6), to reduce the error of the estimated ΔS values, we fitted again the experimental melting curves to the above equation by fixing ΔH at its average value and leaving only ΔS as an adjustable parameter (Table S6). The very good fit of the melting curves, calculated by Eq. 14 using the average ΔH value and the ΔS values from Table S6, to the experimental points is shown in Figure S10.

1.3 Ligand-release kinetics of DNA-based receptors

Ligand-release kinetics for synthetic DNA-based bivalent receptors have been carried out using two triplex-forming DNA receptors (4-nt and 60-nt) and the 11-nt DNA ligand 1 (Figure 3A-B). In the first step, we prepared two different stock solutions containing 10 μM of triplex-forming receptor and 1 μM of 11-nt DNA ligand in the working buffer (PBS buffer, 10 mM $MgCl_2$, at pH 5.5). Before use, each solution was heated to 90°C for 5 min and then allowed to cool to room temperature for 1 h. Then, fresh working buffer has been added in a quartz cuvette to reach a final volume of 1 ml, and it has been heated at a specific temperature which has been selected to induce a controlled release of 11-nt DNA ligand 1. Specifically, for the 4-nt triplex-forming DNA receptor we selected 50°C, 55°C, 57°C, 58.5°C, 60°C and 70°C (Figure 3A) while for the 60-nt triplex-forming DNA receptor we used 40°C, 48°C, 50°C, 52°C, 55°C, 60°C and 70°C (Figure 3B). We heated the cuvette for at least 10 min to ensure that the solution can properly reach the programmed temperature. After this, we started to collect continuously the fluorescence signal until we achieved a stable background signal, then, we added 10 μl of the stock solution containing the triplex-forming DNA receptor and the labelled ligand into the cuvette (for a final concentration of triplex receptor of 100 nM and 10 nM of ligand), and we collected the fluorescence signal for 5 minutes until the signal reach a constant value. To estimate the kinetic constant of the release process at the different temperatures for the triplex-forming receptors, the obtained kinetic profiles have been fitted using the following mono exponential equation:

$$\boldsymbol{y} = \boldsymbol{F_0} + \boldsymbol{b}^{(-\boldsymbol{kt})} \qquad \text{(eq. 2)}$$

where F_0 represent the raw fluorescence signal upon the addition of the oligonucleotides in the solution, b describes the growth/decay rate, k is the kinetic constant of the release process, and t is the time (min). In Table S4 are reported *k* values associated to each triplex-forming receptor. Finally, the raw fluorescence signal has been converted to % ligand released by using the upper

and lower baselines estimated during the analysis of the melting curves (Figure 2B).

Release kinetics for ATP-binding aptamers have been carried out using two variants (4-nt and 70-nt; Figure S12). We followed the same procedure used for the triplex-forming receptors and described previously. Specifically, we prepared two different stock solutions containing 5 µM of ATP-binding aptamer and 30 mM of ATP, and we used as working buffer 100 mM Tris HCl and 10 mM $MgCl_2$, 3 mM ATP at pH 6.5. For the 4-nt ATP-binding aptamer we selected as temperatures 30°C, 40°C, 55°C, 57°C, 59°C, 62°C, 65°C and 70°C (Figure S7) while for the 70-nt ATP-binding aptamer we used 30°C, 36°C, 40°C, 43°C, 45°C, 47°C, 59°C, and 70°C (Figure S12). Once the solution in the cuvette has reached the programmed temperature, we added 10 µl of the stock solution containing the labelled ATP-binding aptamer and the ATP into the cuvette (for a final concentration of aptamer of 500 nM and 3 mM of ATP ligand). Again, to estimate kinetic constants from kinetic profiles of ATP and the conversion of the raw fluorescence to % ligand release we used the same approach described previously. In Table S7 are reported kt (min^{-1}) values associated to ATP binding aptamer variants.

1. 4 Cyclic temperature-jump experiments

Cyclic temperature-jump experiments for triplex-forming DNA receptors (Figure 3C) were performed by mixing in the same solution (10 mL) two receptors (4-nt and 60-nt; 100 nM each) with their corresponding labeled 11-nt DNA ligands (10 nM each). Specifically, the first one (i.e., 11-nt DNA ligand 1) can bind the receptor with a linker of 4-nt and it is labeled with AF488/BHQ-1 pair; the second one (i.e., 11-nt DNA ligand 2) can bind the 60-nt triplex-forming receptor and it is labeled with A-680/BHQ-2 pair. Time course experiments have been performed in working buffer (PBS buffer, 10 mM $MgCl_2$, at pH 5.5) using a final volume of 1 mL in a quartz cuvette,

and the raw fluorescence signals were collected by exciting and acquiring at two different wavelengths (490 ±5 nm/520 ±5 nm and 680 ±5 nm/702 ±5 nm), simultaneously. Firstly, 1 mL of the solution containing the receptors and the DNA ligands was added in the cuvette and the fluorescence signals were continuously recorded at 30°C for 10 mins. At the same time, 2 ml of the same solution was pre-incubated in a test tube and heated in a dry block thermostat (Biosan Bio TDB-100) set at the same temperature. Once the fluorescence signals were acquired at 30 °C, a new empty cuvette was pre-heated to the next temperature (50 °C) and after 10 minutes (necessary for the cuvette to heat up) the previous solution present in the thermostat (at the temperature of 30 °C) was quickly loaded into the hotter cuvette and the signal has been recorded for 10 mins. Using the same procedure, the fluorescence signals relating to the next temperature-jumps from 50 to 70 °C, from 70 to 50 °C and from 50 to 30 °C were obtained and recorded. Finally, the raw fluorescence signals have been converted to % ligand released by using the upper and lower baselines, estimated through analysis of the melting curves of the two triplex-forming receptors in the presence of 11-nt DNA ligands, and Eq. 1 in the main text.

Cyclic temperature-jump experiments for ATP-binding aptamers (Figure 4D) were performed by mixing in the same solution two different variants (4-nt ATP binding aptamer labeled with A-488/BHQ-1 pair, and 40-nt ATP binding aptamer labeled with Cy5.5/BHQ-2 pair; 50 nM each) in the presence of 3 mM ATP. Time course experiments have been performed in 100 mM Tris HCl, 10 mM $MgCl_2$, at pH 6.5, in a quartz cuvette with a final volume of 1 mL, and the fluorescence signals were obtained by exciting and acquiring at two different wavelengths (490 ±5 nm/520 ±5 nm and 673 ±5 nm/707 ±5 nm), simultaneously. The temperature jumps from 30 to 52°C, from 52 to 70 °C, from 70 to 52 °C, and from 52 to 30 °C were obtained using the same procedure described above. Finally, the raw fluorescence signals have been converted to % ligand released by using

the upper and lower baselines, estimated through analysis of the melting curves of the two aptamers in the presence of ATP molecules, and Eq. 1 in the main text.

1.5 Load/release cycle

Load/release cycle experiments were performed using a PCR real-time Stratagene Mx3005SP (Agilent Technologies) where the excitation wavelength was fixed at 490 nm and the acquisition wavelength was fixed at 517 nm. To perform load/release cycle experiments for triplex-forming DNA receptors (Figure 3D-E) a solution containing a fixed concentration of the receptor (4-nt and 60-nt at 500 nM) and the 11-nt DNA ligand 1 (50 nM) was prepared in PBS buffer (10 mM phosphate buffer solution, 137 mM NaCl, 2.7 mM KCl) with10 mM $MgCl_2$, at pH 5.5. 100 µl of this solution was added in three wells of a 96-well PCR plastic plate. Then, the instrument was programmed to execute two different temperature cycles. In the first one, the temperature was held for 30 seconds at 25°C before the fluorescence signal was recorded. After this step, the temperature was increased to 55°C (with a ramp of □0.8°C/s), held for 30 seconds before the fluorescence signal was recorded. Then, the temperature was decreased to the initial one (with a ramp of □0.5°C/s), held for 30 seconds and the fluorescence signal recorded. This was repeated for ten complete cycles. In the second cycle, the temperature was held for 30 seconds at 55°C and next increased to 65 °C. Again, this temperature gradient was repeated back and forward for ten complete cycles. Finally, the raw fluorescence signal was converted to % ligand release by using the upper and lower baselines, estimated through analysis of the melting curves performed in the PCR instrument using the two triplex-forming receptors with the 11-nt DNA ligand 1, and Eq. 1 in the main text.

To perform load/release cycle experiments for ATP-biding aptamers (Figure 4E) we used the same

procedure described above. Specifically, a solution containing a fixed concentration of the aptamer variant (4-nt and 40-nt at 500 nM) and ATP (3 mM) was prepared in 100 mM Tris HCl, 10 mM $MgCl_2$, at pH 6.5, and loaded in the wells. Two different temperature cycles were programmed. In the first one, the temperature was held for 30 seconds at 35°C and increased to 50°C. Then, the temperature was decreased to the initial one, held for 30 seconds and the fluorescence signal recorded. This was repeated for ten complete cycles. In the second cycle, the temperature was held for 30 seconds at 50°C and next increased to 70 °C. Again, this temperature gradient was repeated back and forward for ten complete cycles. The raw fluorescence signal was converted to % ligand release by using the upper and lower baselines, estimated through analysis of the melting curves performed in the PCR instrument using the two aptamer variants in the presence of ATP molecules, and Eq. 1 in the main text.

II – SUPPORTING FIGURES

Linker length (nt)	$T_{50\%}$ release (°C)
4	58.4 ± 0.5
6	56.7 ± 0.5
15	55.9 ± 0.5
30	55.1 ± 0.5
60	50.7 ± 0.5
90	51.0 ± 0.5
4 (Random TFO)	45.3 ± 0.5
60 (Random TFO)	45.7 ± 0.5

Table S1. In the table are reported all the $T_{50\%}$ (°C) values associated with each triplex-forming DNA receptor obtained from the melting curves shown in Figure 2, panel B.

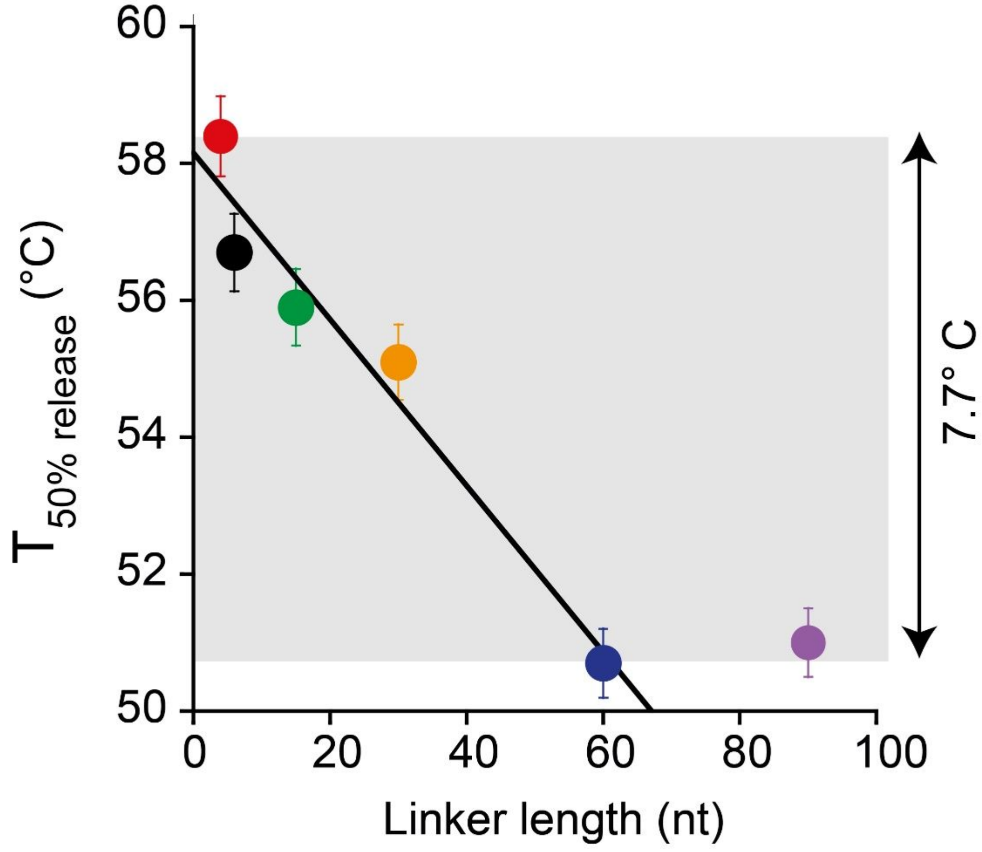

Figure S1. $T_{50\%}$ values as a function of the linker length. By increasing the poly(T) linker length from 4-nt to 60-nt we can modulate the $T_{50\%}$ values displayed by the receptors within a temperature window of 7.7 ± 1°C. Moreover, fitting such values with a linear equation (Y= 58,095 (± 0,48) – 0,11632(±0.0155) · X) allows estimating how each thymine added in the linker shifts the T50% value.

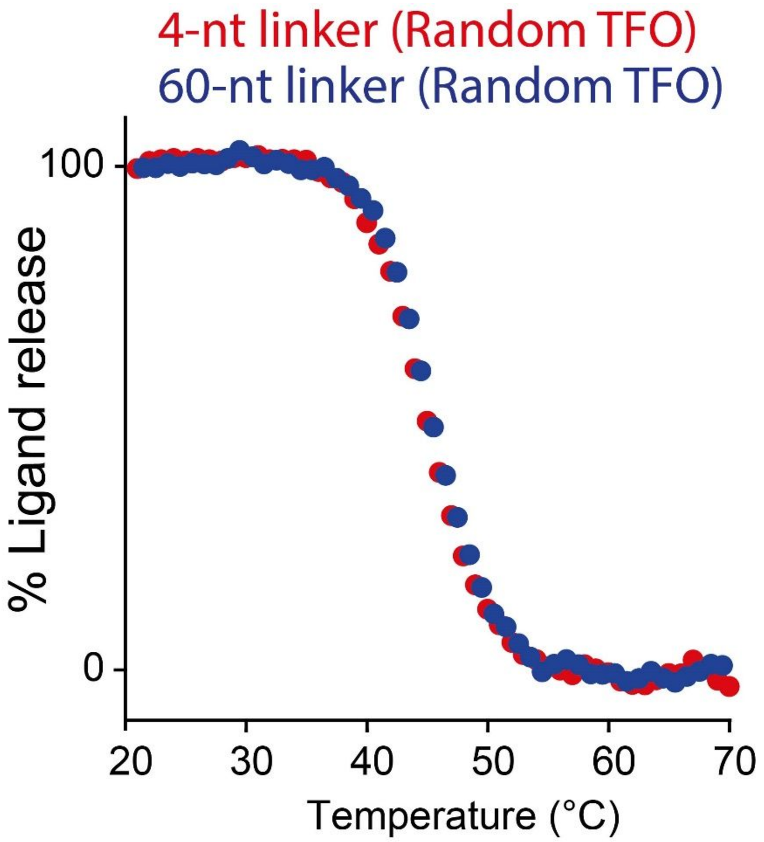

Figure S2. Melting curve experiments of monovalent triplex-forming DNA receptors in which the triplex-forming portion is replaced with a random sequence unable to form the triplex structure. We tested two receptors (100 nM) with a loop of 4-nt (red curve) and 60-nt (blue curve) and a ligand of 11 mer (10 nM).

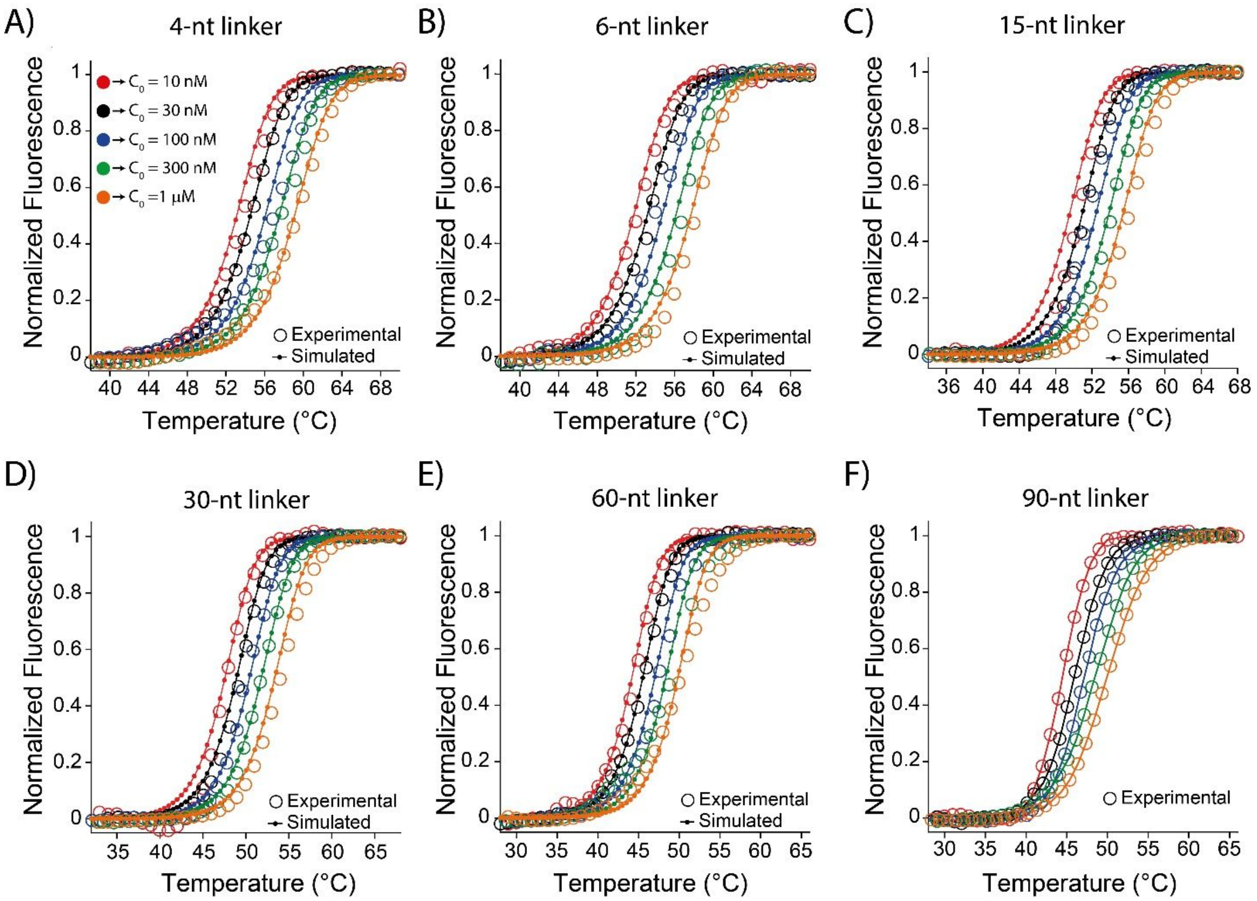

Figure S3. Melting curve experiments performed at different equimolar concentrations (C_0) of triplex-forming DNA receptors and 11-nt target and simulated melting curves obtained from the theoretical model (Eq. S9). Using the estimated $T_{50\%}$ values it is possible estimate the enthalpic and entropic contribution through the analysis of $1/T_{50\%}$ versus $\ln(C_0)$ plot (Figure 2C), and this set of thermodynamic values has been used to predict the melting curves by the theoretical model (Eq. S9).

4-nt linker	$T_{50\%}$ release (°C) MEASURED	PREDICTED
C_0 = 10 nM	54.0 ± 0.5	53.1
C_0 = 30 nM	54.9 ± 0.5	54.6
C_0 = 100 nM	55.5 ± 0.5	55.9
C_0 = 300 nM	58.1 ± 0.5	57.5
C_0 = 1 μM	59.5 ± 0.5	59.0

6-nt linker	$T_{50\%}$ release (°C) MEASURED	PREDICTED
C_0 = 10 nM	52.1 ± 0.5	51.8
C_0 = 30 nM	53.6 ± 0.5	53.1
C_0 = 100 nM	54.5 ± 0.5	54.7
C_0 = 300 nM	56.6 ± 0.5	56.1
C_0 = 1 μM	58.2 ± 0.5	57.7

15-nt linker	$T_{50\%}$ release (°C) MEASURED	PREDICTED
C_0 = 10 nM	50.5 ± 0.5	49.6
C_0 = 30 nM	51.3 ± 0.5	50.9
C_0 = 100 nM	51.9 ± 0.5	52.4
C_0 = 300 nM	54.1 ± 0.5	53.9
C_0 = 1 μM	56.1 ± 0.5	55.4

30-nt linker	$T_{50\%}$ release (°C) MEASURED	PREDICTED
C_0 = 10 nM	47.8 ± 0.5	47.6
C_0 = 30 nM	49.4 ± 0.5	49.1
C_0 = 100 nM	50.2 ± 0.5	50.5
C_0 = 300 nM	51.9 ± 0.5	51.9
C_0 = 1 μM	54.2 ± 0.5	53.4

60-nt linker	$T_{50\%}$ release (°C) MEASURED	PREDICTED
C_0 = 10 nM	44.3 ± 0.5	44.3
C_0 = 30 nM	45.6 ± 0.5	45.5
C_0 = 100 nM	47.5 ± 0.5	47.1
C_0 = 300 nM	48.9 ± 0.5	48.4
C_0 = 1 μM	50.1 ± 0.5	49.9

90-nt linker	$T_{50\%}$ release (°C) MEASURED
C_0 = 10 nM	44.5 ± 0.5
C_0 = 30 nM	45.9 ± 0.5
C_0 = 100 nM	47.3 ± 0.5
C_0 = 300 nM	48.6 ± 0.5
C_0 = 1 μM	50.2 ± 0.5

Table S2. $T_{50\%}$ (°C) values associated with each triplex-forming DNA receptor and estimated at different concentrations (C_0) and the values estimated from the simulated melting curves through the theoretical model (Eq. S9). These values are obtained from the melting curves shown in Figure S3.

Linker length (nt)	Free ΔH (kcal*mol^{-1})	ΔS (cal*mol^{-1}*K)
4	175.9 ± 8.8	470.5 ± 1.0
6	162.6 ± 8.2	472.6 ± 1.0
15	173.3 ± 8.7	476.1 ± 1.0
30	158.7 ± 8.0	479.3 ± 1.0
60	158.8 ± 8.0	484.8 ± 1.0
90	166.4 ± 8.0	484.7 ± 1.0
	fixed ΔH = 165.9 ± 8.3	

Table S3. Thermodynamic parameters (Free ΔH, fixed ΔH and ΔS) associated with each triplex-forming DNA receptor estimated through thermodynamic analysis (Figure 2C).

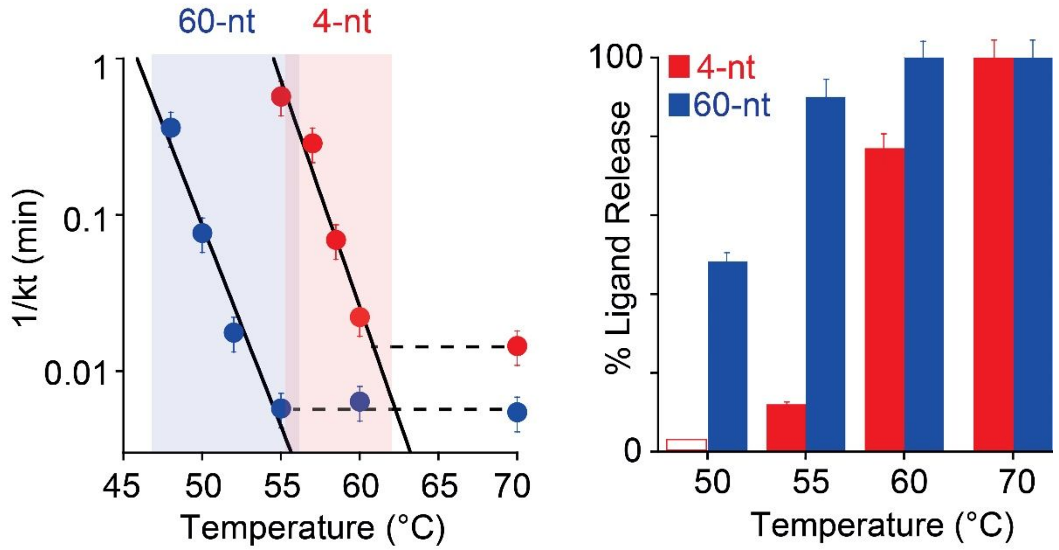

Figure S4. (Left) 1/kt vs temperature plot for the two DNA receptors. (Right) Percentage of ligand release for the two receptors at 4 representative temperatures.

Triplex-forming receptor (4-nt)		Triplex-forming receptor (60-nt)	
Temperature (°C)	kt (min^{-1})	Temperature (°C)	kt (min^{-1})
50	-	40	-
55	1.74 ± 0.08	48	2.75 ± 0.11
57	3.47 ± 0.28	50	13.06 ± 0.37
58.5	14.42 ± 0.37	52	56.65 ± 2.04
60	44.99 ± 6.32	55	173.12 ± 8.10
70	69.08 ± 2.54	60	156.92 ± 8.11
		70	183.65 ± 8.58

Table S4. Kinetic constants estimated for the release of the ligand associated with each triplex-forming DNA receptor at different temperatures.

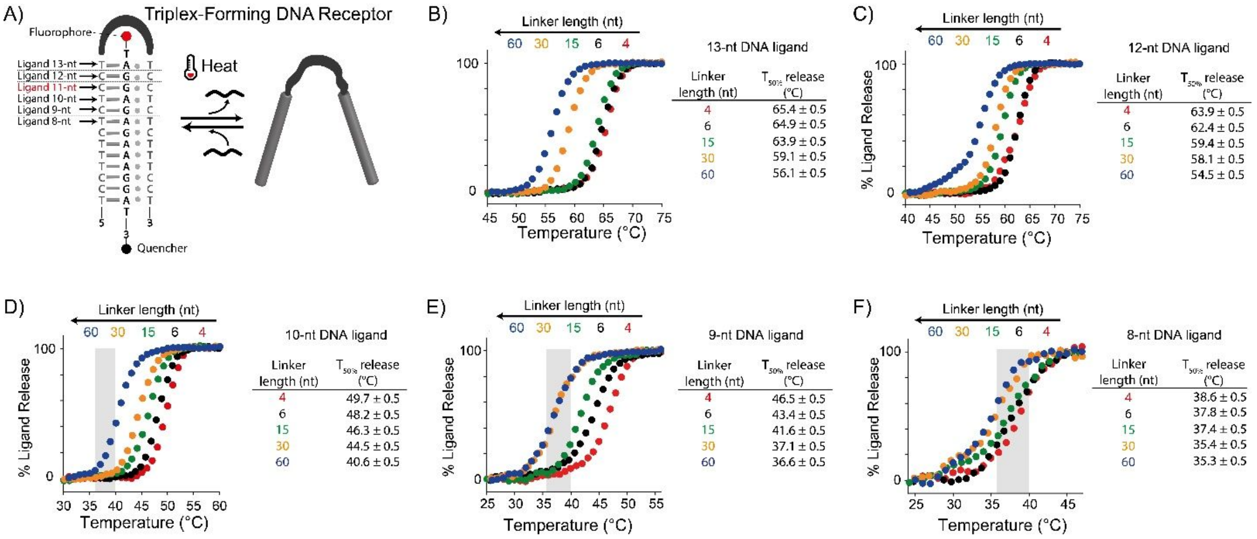

Figure S5. Triplex-forming DNA-based receptors can fully load and release ligands of different lengths. (A) Scheme of the bivalent molecular receptor in which the sequences of the two binding domains and labeled DNA ligands are described. (B-F) Melting curves of the set of triplex-forming DNA receptors sharing the same binding domains with complementary DNA ligands of different lengths and their estimated $T_{50\%}$ values.

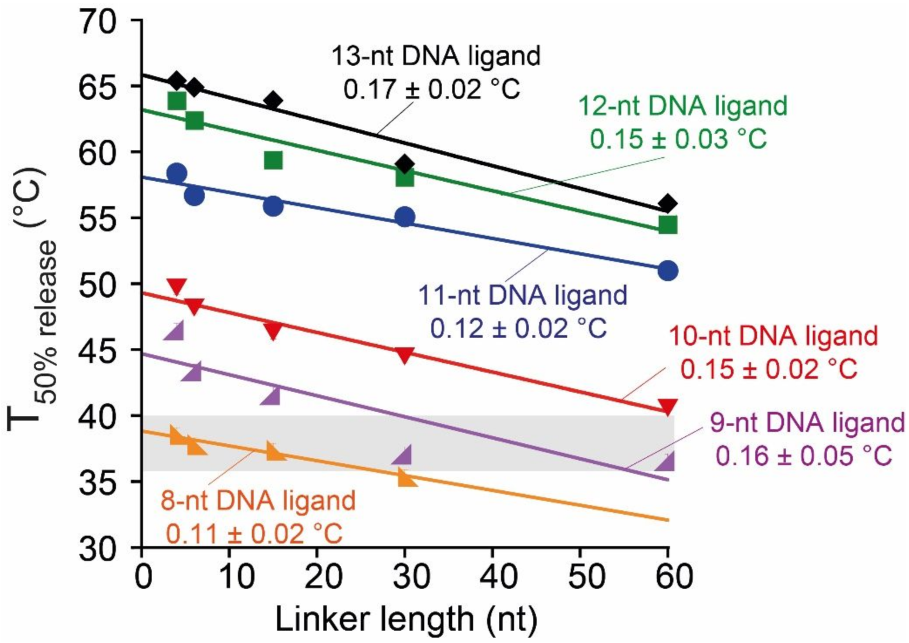

Figure S6. Plot of $T_{50\%}$ values as a function of the linker length for the triplex-forming DNA receptors and the different DNA ligands tested. By increasing the poly(T) linker length from 4-nt to 60-nt we can modulate the $T_{50\%}$ values displayed by the receptors for all DNA ligands. Of note, varying the length of the DNA ligand we can shift the temperature range of the load/release process towards higher or lower temperature values. Fitting such values with a linear equation allows estimating how each thymine added in the linker shifts the $T_{50\%}$ value and this effect results similarly for the set of DNA ligands tested.

A)

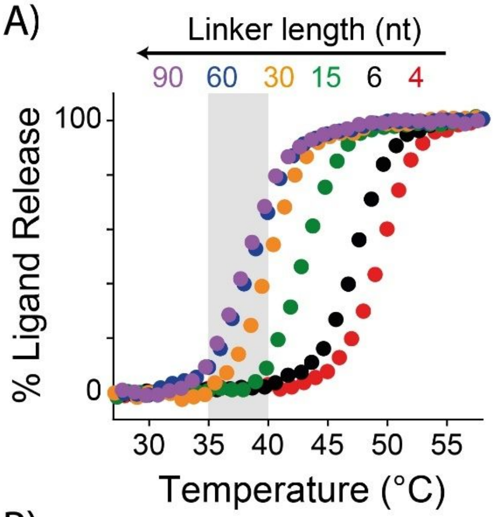

Linker length (nt)	$T_{50\%}$ release (°C)	
	No Mg^{2+}	10 mM Mg^{2+}
4	49.3 ± 0.5	58.4 ± 0.5
6	47.3 ± 0.5	56.7 ± 0.5
15	43.1 ± 0.5	55.9 ± 0.5
30	40.2 ± 0.5	55.1 ± 0.5
60	38.8 ± 0.5	50.7 ± 0.5
90	38.3 ± 0.5	51.0 ± 0.5

B)

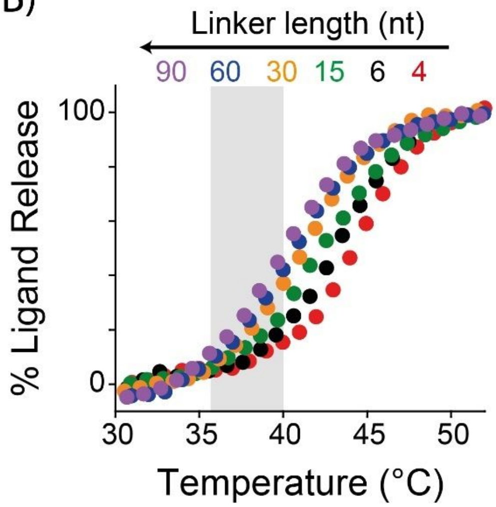

Linker length (nt)	$T_{50\%}$ release (°C)	
	pH 6.5	pH 5.5
4	44.2 ± 0.5	58.4 ± 0.5
6	43.2 ± 0.5	56.7 ± 0.5
15	42.3 ± 0.5	55.9 ± 0.5
30	41.2 ± 0.5	55.1 ± 0.5
60	40.6 ± 0.5	50.7 ± 0.5
90	40.2 ± 0.5	51.0 ± 0.5

Figure S7. Effect of pH and magnesium ions on the load/release temperature. (A) Melting experiments performed in absence of Mg^{2+} ions and the associated $T_{50\%}$ values for each receptor. (B) Melting experiments performed at pH 6.5 (instead of 5.5) and the associated $T_{50\%}$ values for each receptor.

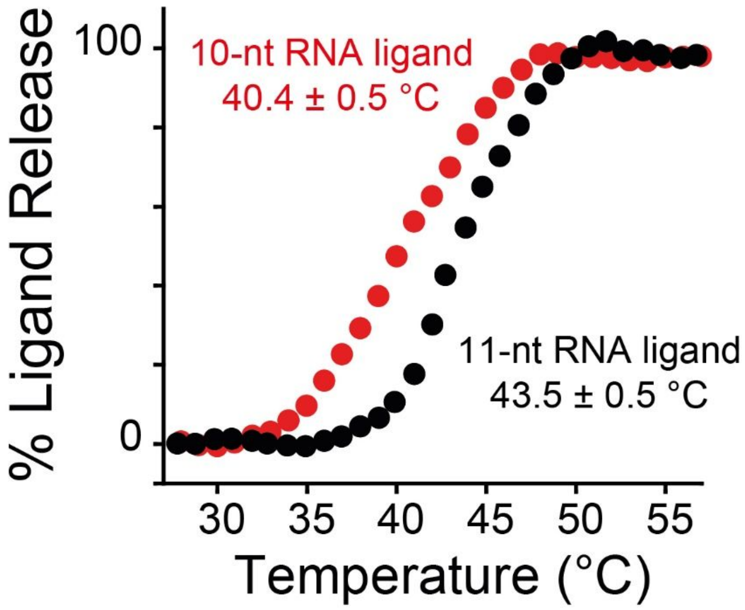

Figure S8. The triplex-forming DNA receptors can recognize and bind RNA ligands. Melting experiments performed using the receptor with a linker length of 4-nt labeled at its two ends (ATTO495-BHQ1) and RNA ligands of 11-nt and 10-nt (in PBS buffer, 10 mM $MgCl_2$ at pH 5.5 with a temperature ramp of 1 °C·min^{-1}).

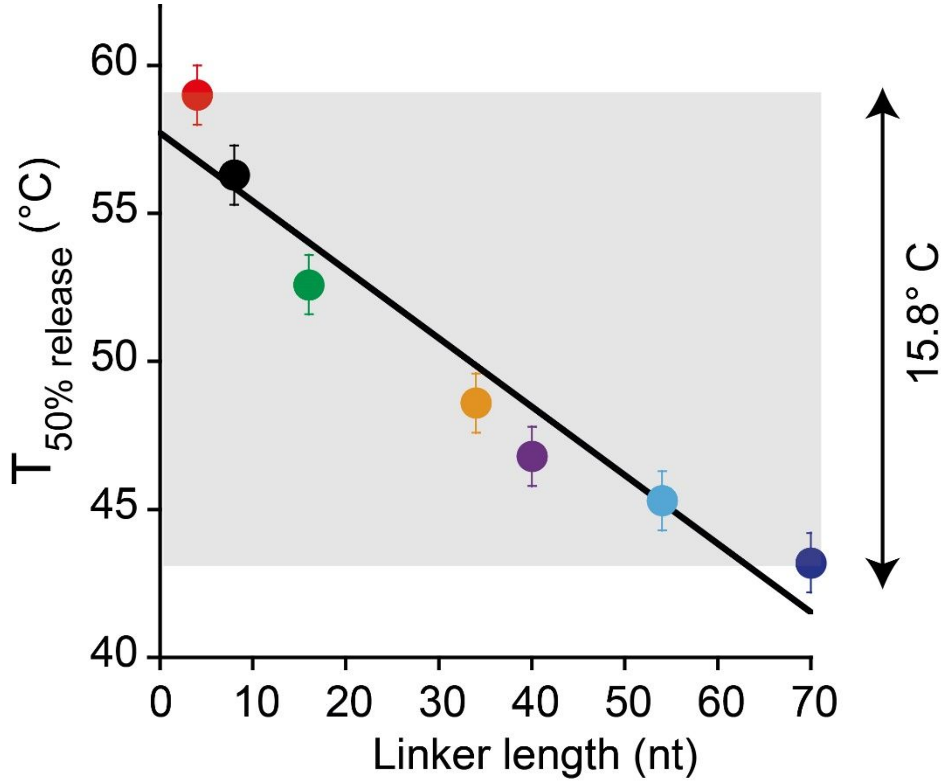

Figure S9. Plot of $T_{50\%}$ values as a function of the linker length for the ATP-binding aptamers. By increasing the poly(T) linker length from 4-nt to 70-nt we can modulate the $T_{50\%}$ values displayed by the aptamer variants within a temperature window of 15.8 ± 1°C. Moreover, fitting such values with a linear equation (Y= 57,638 (± 1,15) – 0,23(±0.03) · X) allows estimating how each thymine added in the linker shifts the T50% value.

Linker length (nt)	$T_{50\%}$ release (°C)
Native	61.7 ± 0.5
4	59.0 ± 0.5
8	56.3 ± 0.5
16	52.6 ± 0.5
32	48.6 ± 0.5
40	46.8 ± 0.5
54	45.3 ± 0.5
70	43.2 ± 0.5

Table S5. In the table are reported all the $T_{50\%}$ (°C) values associated with each ATP-binding aptamer variant obtained from the melting curves shown in Figure 4, panel B.

Linker length (nt)	Free ΔH (kcal*mol^{-1})	ΔS (cal*mol^{-1}*K)
4	73.4 ± 2.6	201.6 ± 1.0
8	78.7 ± 2.4	203.4 ± 1.0
16	69.1 ± 2.8	206.0 ± 1.0
32	74.2 ± 2.4	208.9 ± 1.0
40	76.7 ± 3.0	210.2 ± 1.0
54	75.8 ± 2.4	211.3 ± 1.0
70	74.0 ± 4.4	212.8 ± 1.0

fixed ΔH= 74.6 ± 3.0

Table S6. In the table are reported all the thermodynamic values (ΔH, fixed ΔH and ΔS) associated with each ATP-binding aptamer variant obtained through the analysis of melting experiments using the theoretical model (see SI for further details).

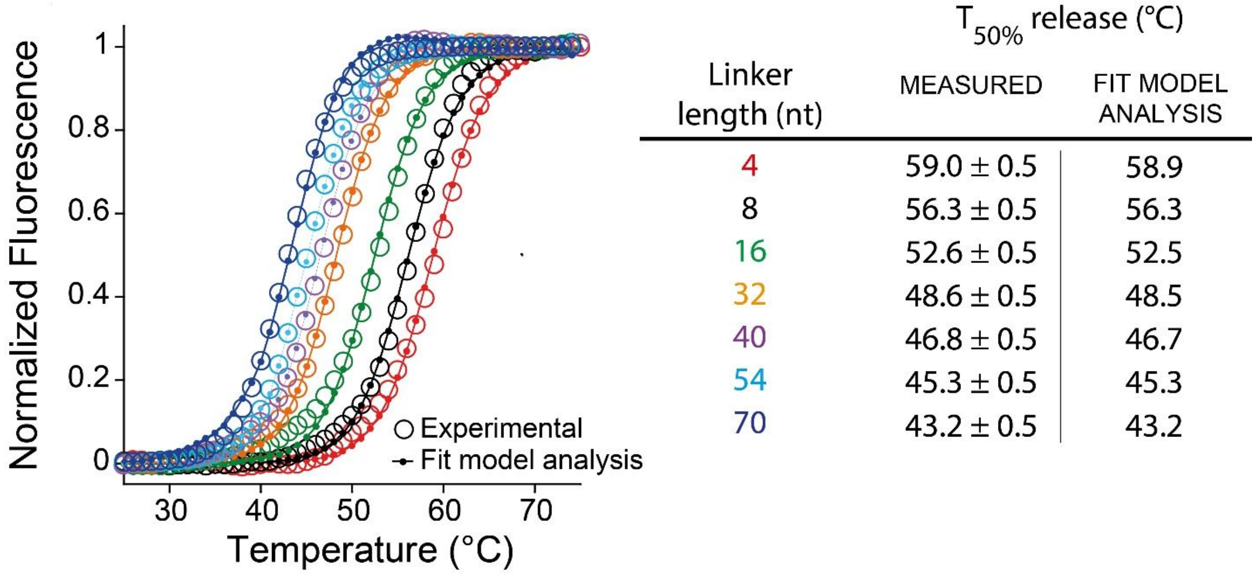

Linker length (nt)	$T_{50\%}$ release (°C) MEASURED	$T_{50\%}$ release (°C) FIT MODEL ANALYSIS
4	59.0 ± 0.5	58.9
8	56.3 ± 0.5	56.3
16	52.6 ± 0.5	52.5
32	48.6 ± 0.5	48.5
40	46.8 ± 0.5	46.7
54	45.3 ± 0.5	45.3
70	43.2 ± 0.5	43.2

Figure S10. Thermodynamic analysis of the melting curves of ATP-binding receptors with different linker lengths obtained from the developed theoretical model (Eq. S14). Using the average ΔH value calculated from a first fitting analysis (Table S6), we precisely estimated the entropic content for each receptor finding a linear correlation between T50% values and the obtained entropy values (Figure 4C).

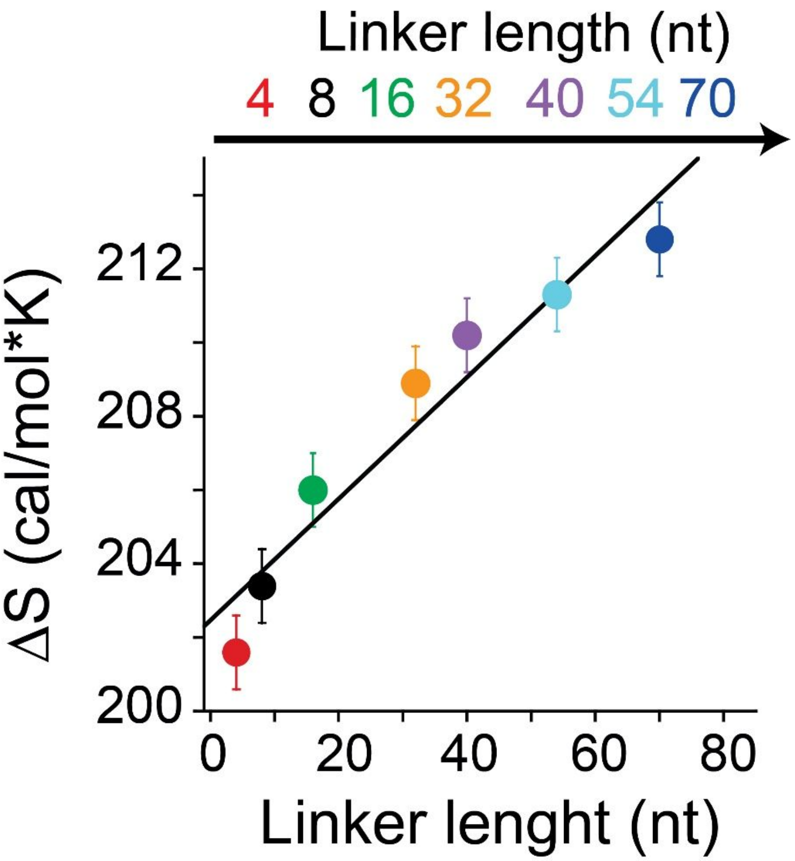

Figure S11. Plot of estimated ΔS vs linker length for the set of aptamer receptors used.

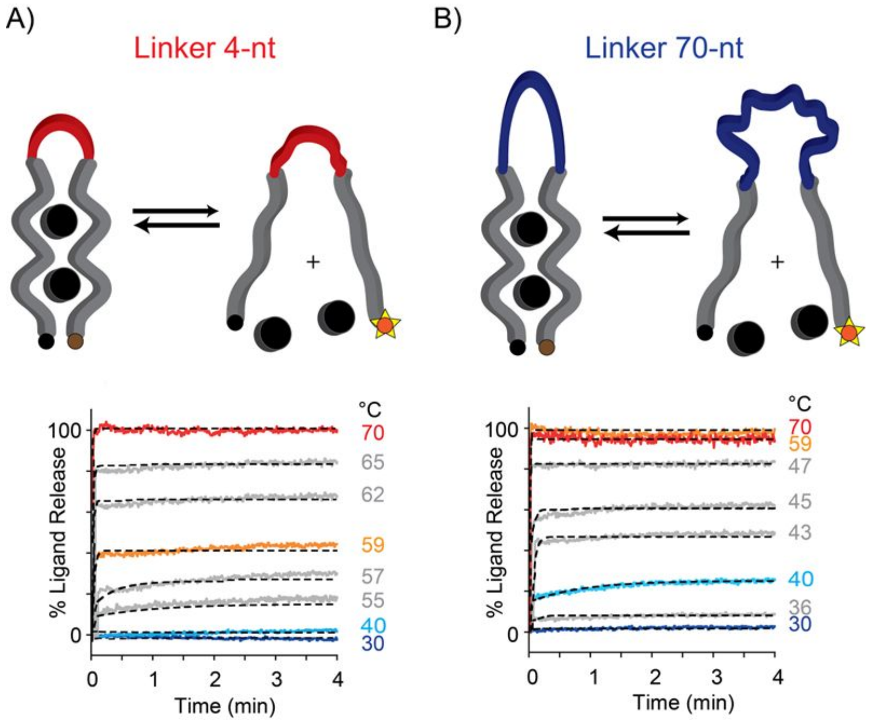

Figure S12. Release kinetics of ATP-binding aptamers with a 4-nt and 70-nt linker domain monitored through temperature jumps. The jumps were performed from an initial temperature of 25 °C and programmed to reach different final temperatures (indicated). Four representative temperatures (30, 40, 59 and 70°C) were coloured for better comparison.

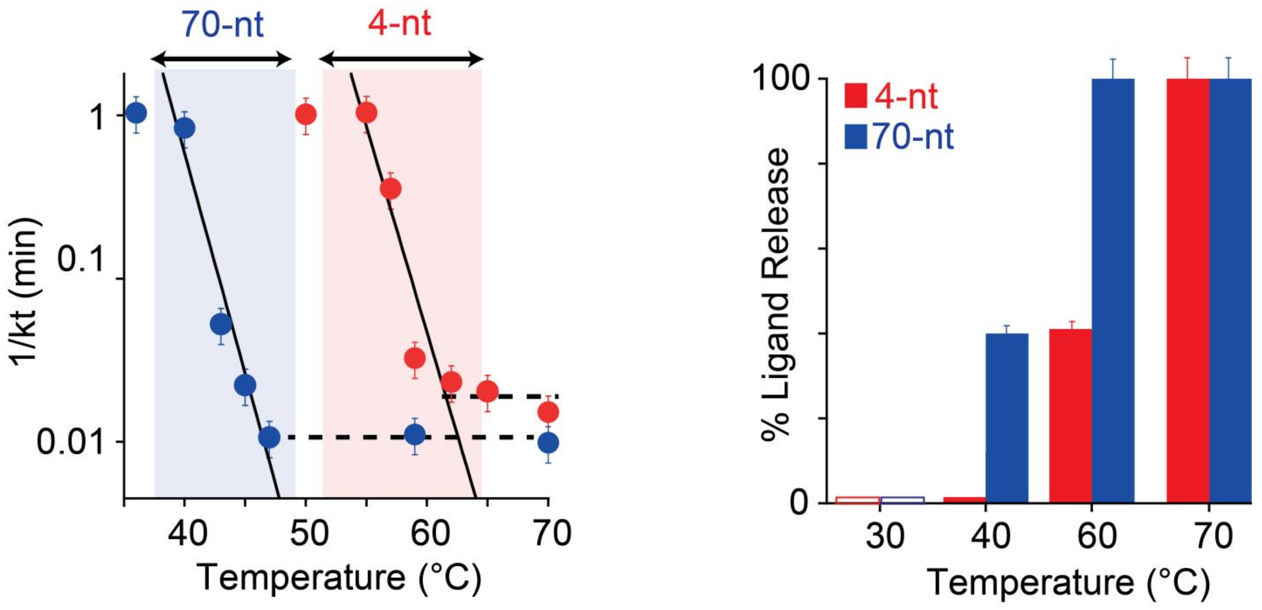

Figure S13. (left) 1/kt vs temperature plot for the two aptamer variants. (right) Percentages of ATP released for the two aptamers at 4 representative temperatures.

ATP binding aptamer (4-nt)		ATP binding aptamer (70-nt)	
Temperature (°C)	kt (min^{-1})	Temperature (°C)	kt (min^{-1})
30	-	30	-
40	-	36	0.96 ± 0.07
55	0.96 ± 0.06	40	1.19 ± 0.08
57	2.80 ± 0.20	43	19.31 ± 0.45
59	30.70 ± 1.43	45	44.90 ± 1.52
62	42.88 ± 1.54	47	93.98 ± 8.49
65	49.05 ± 1.55	59	89.97 ± 4.08
70	65.64 ± 1.42	70	100.91 ± 4.37

Table S7. Kinetic constants estimated for the release of the ligand associated with each ATP-binding aptamer at different temperatures.

III – REFRENCES

(1) Puglisi, J. D.; Tinoco, I. Absorbance Melting Curves of RNA. Methods Enzymol. 1989, 180, 304–325.

(2) Mergny, J. L.; Lacroix, L. Analysis of Thermal Melting Curves. Oligonucleotides 2003, 13 (6), 515–537.

(3) Sigma-Aldrich, URL: https://www.sigmaaldrich.com/deepweb/assets/sigmaaldrich/product/documents/786/464/a3377pis.pdf (accessed 07/24/2022).

(4) Mariottini, D.; Idili, A.; Nijenhuis, M. A. D.; De Greef, T. F. A.; Ricci, F. DNA-Based Nanodevices Controlled by Purely Entropic Linker Domains. J. Am. Chem. Soc. 2018, 140 (44), 14725–14734.
